# Interfacial Photoelectrochemistry in Organic Synthesis

**DOI:** 10.1002/anie.202424300

**Published:** 2025-09-21

**Authors:** Gabriel Chan, Daria Corsi, Oleksandr Savateev, Paolo Giusto, Joshua P. Barham

**Affiliations:** ^1^ Colloid Chemistry Department Max Planck Institute of Colloids and Interfaces Am Mühlenberg 1 14476 Potsdam Germany; ^2^ Institute of Organic Chemistry University of Regensburg Universitätsstr. 31 93053 Regensburg Germany; ^3^ Department of Chemistry The Chinese University of Hong Kong Shatin New Territories Hong Kong China; ^4^ Department of Pure & Applied Chemistry University of Strathclyde 295 Cathedral Street Glasgow G1 1XL UK

**Keywords:** Dye‐sensitized, Interfacial photoelectrochemistry, Photoelectrocatalysis, Photoelectrode, Semiconductor

## Abstract

Photoelectrodes have traditionally enjoyed widespread attention as heterogeneous catalysts for the activation of water and CO_2_ in energy research, while photoelectrochemistry with homogeneous molecular catalysts dominates the activations of more complex molecules in organic synthesis. Nonetheless, interfacial photoelectrochemistry (iPEC) offers great benefits to organic synthesis, including catalyst cost‐efficiency, reusability, and stability. This review aims i) for a comprehensive collection of historical and recent examples of iPEC and ii) to present the field in manner and language accessible to synthetic chemists. Conceptual comparisons from photoelectrodes to homogeneous (electro‐activated) photocatalysts to dye‐sensitized photoelectrodes will be drawn, with advantages and limitations of each catalyst archetype discussed. Surface techniques for fabrication of photoelectrodes will be introduced. Future semiconductor photoelectrode materials, substrate targets, and conceptual challenges in the field will be highlighted.

## Graphical abstract

Heterogeneous photoelectrodes are widespread for activations of H_2_O and CO_2_, while homogeneous photoelectrochemistry with molecular catalysts dominates complex molecule activations in synthesis. Nonetheless, interfacial photoelectrochemistry (iPEC) offers great potential for synthesis, including catalyst cost‐efficiency, reusability, and stability. This review comprehensively presents the field in a manner accessible to synthetic chemists, including surface techniques for photoelectrode fabrication.

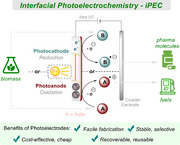

## 1 Introduction

With the constantly intensifying hazard of global warming and environmental concerns, transforming chemistry into a more sustainable field has been of utmost importance in recent decades. Chemical catalysis has proven itself as a viable solution to address problems in chemical synthesis by lowering activation energies, easing reaction conditions, and increasing product selectivities. Nevertheless, the catalysts themselves also pose other problems—they often contain precious transition metals (TMs) and organic ligands that can be scarce and expensive, which pose questions concerning their sustainability. Not only does the procurement and synthesis of such materials create additional threats to the environment, the fact is that many of these catalysts are not recoverable, reusable, or recyclable. This has pushed scientists to search for other possible solutions, such as revisiting old research fields with a fresh perspective and devising new methods to achieve reaction goals in a more efficient manner.

In the fields of organic synthesis and small molecule functionalization, two catalytic approaches driven by visible light or electricity as sustainable energy sources—photoredox catalysis (PRC) and synthetic organic electrochemistry (SOE)—have become centers of attention.^[^ ^1^, ^2^ ^]^ While heterogeneous PRC and SOE often deploy transition metal‐based photocatalysts and electrodes, they are recoverable; as opposed to homogeneous PRC, where transition metal complexes require solvent‐intensive conditions and potentially require multiple downstream steps to recover. Both PRC and SOE utilize a process called single electron transfer (SET), which was first described by Faraday in 1834.^[^ ^3^ ^]^ Chemists' attempts to synthesize complex molecules by SET date back as early as the 19^th^ century, such as synthesis of ethane from acetate.^[^ ^4^, ^5^, ^6^ ^]^ Traditionally, SET processes are induced by exogenous stoichiometric chemical one‐electron oxidants or reductants added to the reaction system, where a single electron is transferred from the electron donor to the electron acceptor.^[^ ^7^ ^]^ It was later discovered that SET can also be induced by the combination of solar and electrical energy—photoelectrochemistry (PEC)—with protons or water as electron and hole sources, substituting for sacrificial organic redox agents and thus decreasing the amount of chemical waste produced.^[^ ^8^ ^]^

**Figure 1:**
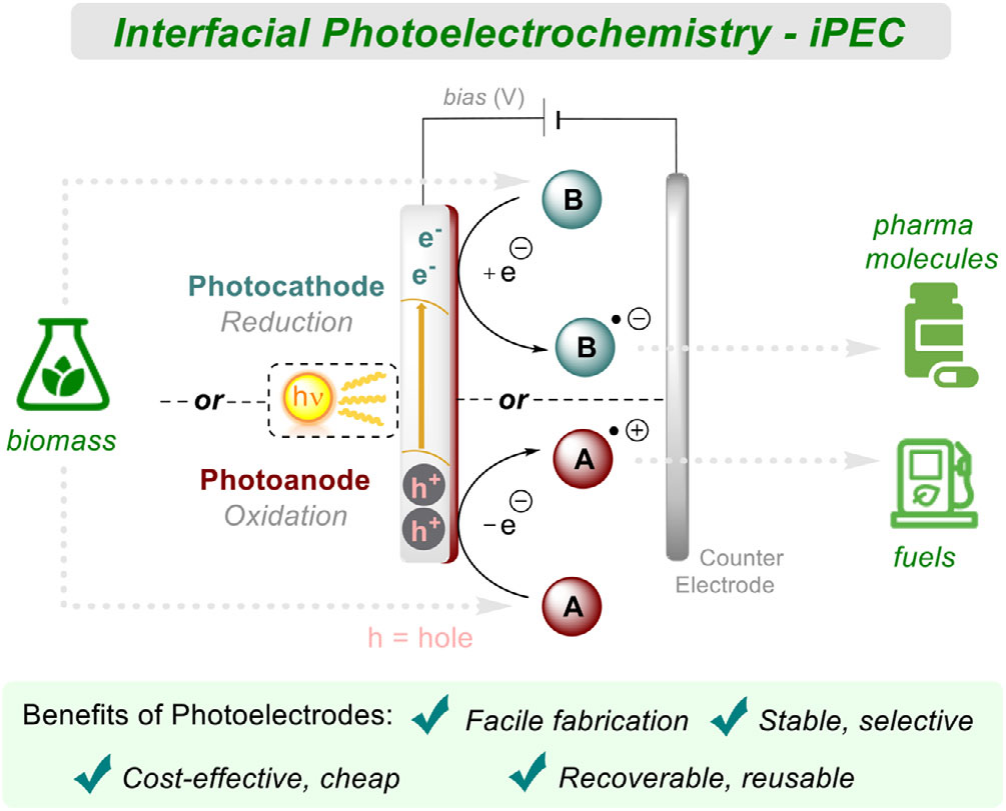
Interfacial photoelectrochemistry.

In this review, we depict a general overview and promising results for different categories of interfacial photoelectrocatalytic reactions. Contrasting with molecular PEC that involves light‐harvesting homogeneous catalysts (often coined by practitioners as electro‐activated/electrochemically‐mediated PhotoRedox Catalysis (e‐PRC) or “electrophotocatalysis” (EPC)) or reactants, this review's focus is on heterogeneous photoelectrodes (photoelectrocatalysis), covering their fabrications and applications to organic molecule synthesis or activations. Along these lines, we aim to outline a forecast for more sustainable and active heterogeneous materials. Special regard will be given to covalent materials with high activities toward more challenging and energy‐intensive reactions (for example, direct reductions of CO_2_ and N_2_); not just to produce small molecules like methanol and ammonia, but also for direct incorporation of such reactions to synthesize value‐added organic molecules (Figure 1).

### 1.1 Photoredox Catalysis

Photoredox catalysis (PRC), or more generally ‘photocatalysis’, was recognized as a viable synthetic platform to build or modify organic compounds over a century ago. As early as 1839, Becquerel discovered and utilized a photovoltaic cell to harvest solar energy.^[^ ^9^ ^]^ Then, in the early 20th century, Ciamician provided a systematic and valuable vision on using solar energy as a replacement for fossil fuels to drive the synthesis of organic molecules, due to solar energy being inexhaustible.^[^ ^10^ ^]^ Ciamician was undoubtedly inspired by photosynthesis; the reaction pathway where plants make use of solar energy to convert CO_2_ and water into carbohydrates via chlorophyll (the colored pigment of photosystem II). Indeed, the early “renaissance” of examples of applying PRC in organic synthesis were achieved by using homogeneous transition metal complexes that were found to act in similar fashion (at least conceptually) to chlorophyll in plants, such as the bipyridine complexes of Ru(II) and Ir(III).^[^ ^11^, ^12^, ^13^, ^14^ ^]^ A key advance is that the product molecules are complex small molecules that are far more valuable than H_2_ or electricity. This underlies the potential of PRC in value‐added applications such as the valorization of biomass and the production of pharmaceutical or agrochemical molecules.

Generally, when a material is irradiated by light, the photon energy is absorbed by a chemical species and used to promote an electron from a lower‐lying energy orbital to a higher‐lying energy orbital, resulting in a ground‐state molecule entering an excited state. In non‐photocatalytic species, the energy provided by photons is released almost immediately afterward, either in a radiative or non‐radiative manner, and the molecule returns to the ground state. This is not the case for most photocatalysts. The excited states of common photocatalysts have longer lifetimes, which means that there is enough time for diffusion and reactions with other molecules in solution. After absorption of a photon, relaxation occurs (internal conversion) to the first accessed excited state, often the singlet excited state (S_1_). The fate of S_1_ is highly differentiated. In most of the transition metal complexes with photocatalytic properties, a phenomenon known as intersystem crossing occurs, where S_1_ is transformed into a triplet excited state (T_1_) that enjoys a longer lifetime.^[^ ^15^ ^]^ The triplet state is then the dominant catalytically active state responsible for reactivity. It can then either accept or donate an electron to reactants via SET, depending on the compatibility (matching) of energy levels of the highest occupied molecular orbital (HOMO) and lowest unoccupied molecular orbital (LUMO) of the photocatalyst and the reactant. However, the internal conversion and intersystem crossing pathways consume a fraction of the initial photon energy input, and this penalty narrows the operational range of redox potential of the excited state catalyst.^[^ ^16^ ^]^

Later, the idea was extended to using organic photocatalysts in organic synthesis to eliminate the reliance on transition metal complexes. This class of compounds includes a multitude of polycyclic aromatic compounds that are soluble in organic solvents and absorb photons in the visible spectrum.^[^ ^15^, ^17^, ^18^, ^19^, ^20^ ^]^ Unlike transition metal complexes, which mostly function through T_1_, S_1_ is a valid photocatalytic state of these polycyclic aromatic dye molecules. Lifetimes in the nanosecond range decrease the probability of S_1_ participating in bimolecular reactions, often necessitating higher catalyst loadings versus TM complexes. Nonetheless, the S_1_ states are more potent oxidants and reductants than the corresponding T_1_ states (of, e.g., TM complexes), which theoretically broadens the range of SET reactions possible.^[^ ^15^ ^]^ Each of them has their distinctive HOMO‐LUMO energy levels suitable for different target reactions, and together with their lower cost and straightforward tunability, this means a broad spectrum of reactions can potentially be enabled by deploying different organophotocatalysts.

Unfortunately, molecular photocatalysts also have complexities of their own. Most notably, they are difficult to recover. Catalytic reactions involving molecular photocatalysts are usually run homogeneously due solubility requirements in organic solvents. While homogeneous phase reactions are advantageous in terms of simplicity and mechanistic/analytical accessibility, they posesconsiderable challenges for scientists to extract and recover the catalyst afterward. Moreover, recent studies and reviews have highlighted the propensities for these homogeneous organic photocatalysts to decompose during their photochemical reactions,^[^ ^21^, ^22^, ^23^, ^24^, ^25^, ^26^, ^27^ ^]^ meaning that the photocatalysts added to the reaction may not be the actual active species. While this oftentimes leads to species that are still (photo)catalytically active,^[^ ^23^, ^24^, ^25^, ^27^ ^]^ it presents issues in product separation and clouds the understanding of the catalytic processes, often necessitating isolation of authentic photoactive species to test independently.^[^ ^28^ ^]^ Moreover, molecular photocatalysts can often photobleach under high‐intensity irradiation.^[^ ^29^, ^30^, ^31^ ^]^

Adding to the problems, not all of these molecular catalysts are commercially available. While some of them, like Eosin Y, Rose Bengal, and mesityl acridinium salts (Mes‐Acr^+^), are inexpensive and readily available from chemical companies, many more lie outside of this category and must be custom synthesized. This greatly hinders the possibilities to upscale the reactions to industrial level, though there exist successful endeavors on scale; such as the photoacylation of naphthoquinone with butyraldehyde, the dye‐sensitized photooxygenations of citronellol and 1,5‐dihydroxy‐naphthalene,^[^ ^32^ ^]^ and the photooxidation of artemisinin.^[^ ^33^ ^]^

### 1.2 Semiconductor Materials and Photoelectrochemistry

In order to circumvent the above‐mentioned drawbacks, chemists have simultaneously explored the use of semiconductor materials, such as metal oxides, sulfides, and polymers. Reports of photocatalytic reactions mediated by semiconductors have been reported as early as 1924, where ZnO was used as a photocatalyst to reduce Ag^+^ to Ag and to degrade the dye Prussian blue.^[^ ^34^ ^]^ Afterwards, some other reports of using other semiconductor materials were made,^[^ ^35^, ^36^ ^]^ but they were limited to small‐scale pioneering research due to the lack of incentives.^[^ ^9^ ^]^ A breakthrough was made in 1972, when Fujishima and Honda reported the groundbreaking concept of photo‐assisted water splitting, which highlighted the potential of semiconductors as photocatalysts.^[^ ^37^ ^]^ The water‐splitting reaction, after decades of development, has reached amazing energy and quantum efficiencies,^[^ ^38^, ^39^, ^40^ ^]^ showcasing the potential of such materials. In the last decade, the use of PEC has also been extended to other inorganic transformations.^[^ ^41^, ^42^, ^43^, ^44^ ^]^

In semiconductors, the concepts of HOMO and LUMO are expanded into those of the valence band and the conduction band, where the difference between the energy levels between these two is called the “band gap.” The band gap of semiconductors can be tuned, e.g., by doping the material with different elements, such as carbon, nitrogen, or even metals.^[^ ^45^, ^46^, ^47^ ^]^ Moreover, semiconductor materials are often recyclable and typically come at lower prices when compared to homogeneous transition metal complexes. Compounding their i) chemical and thermal stability, ii) low cost, iii) processable nature, and iv) recyclability, they therefore match more to Ciamician's vision on replacing fossil fuel‐based industrial organic compound production with photochemistry. To date, although the majority of photocatalysts reported for organic synthetic applications in the last two decades are molecular,^[^ ^12^, ^15^ ^]^ there are also numerous examples using semiconductors as photocatalysts in organic synthesis, with a broad scope of reactions reported.^[^ ^48^, ^49^, ^50^, ^51^, ^52^ ^]^

However, this does not mean that semiconductor photocatalysis is the “key to every door” in the field of sustainable organic synthesis. Ultimately, the energy of a single photon is not sufficient to drive energy‐intensive chemical synthesis and transformations, including the synthesis of glucose from CO_2_ and H_2_O; oxidation of alkyl C─H bonds, ethers, and carbonyl compounds; and the reduction of aryl chlorides and aromatic systems.^[^ ^1^ ^]^ While there have been reports on addressing this issue by compiling multiphoton energies, such are mainly limited to organophotocatalysts that mostly require the use of superstoichiometric sacrificial redox agents (particularly, trialkylamines, e.g., 3–5 equivalents of R_3_N).^[^ ^53^, ^54^, ^55^ ^]^ Here, inspired by the well‐developed systems used for water‐splitting, which combines electrochemistry (EC) and photocatalysis, the idea of combining PRC and SOE, dubbed “synthetic photoelectrochemistry” (PEC), came into play. According to the first full review of synthetic PEC, the field was classified into three subtypes: (i) electrochemically mediated photoredox catalysis (e‐PRC), (ii) decoupled photoelectrochemistry (dPEC), and (iii) interfacial photoelectrochemistry (iPEC).^[^ ^1^ ^]^ This review will focus on type (iii), as the other two types generally refer to light‐absorbing homogeneous catalysts or reactants where the roles of electrochemical and photochemical steps are distinct, even within the same catalytic cycle. In iPEC, the photoelectrode plays both roles as a single heterogeneous surface catalyst. As the previous reviews regarding iPEC in organic chemistry often also include a mixture of examples and concepts of e‐PRC and dPEC,^[^ ^1^, ^56^, ^57^ ^]^ this review aims to provide a uniform, comprehensive summary of achievements and challenges in heterogeneous photoelectrodes for organic synthetic applications and to make the topic more accessible to the synthetic chemist. The basic concepts of iPEC, cell setup and components, the preparation of electrodes, as well as reported applications of semiconductor photoelectrode‐based iPEC in organic synthesis will be discussed. The broad aim of this review, as well as to summarize key progress, challenges, and opportunities in the field of iPEC, is to “demystify” the aspects of this technique and to make it more accessible for practicing synthetic chemists.

## 2 iPEC—Principles, Components, Fabrication of Photoelectrodes

**Figure 2:**
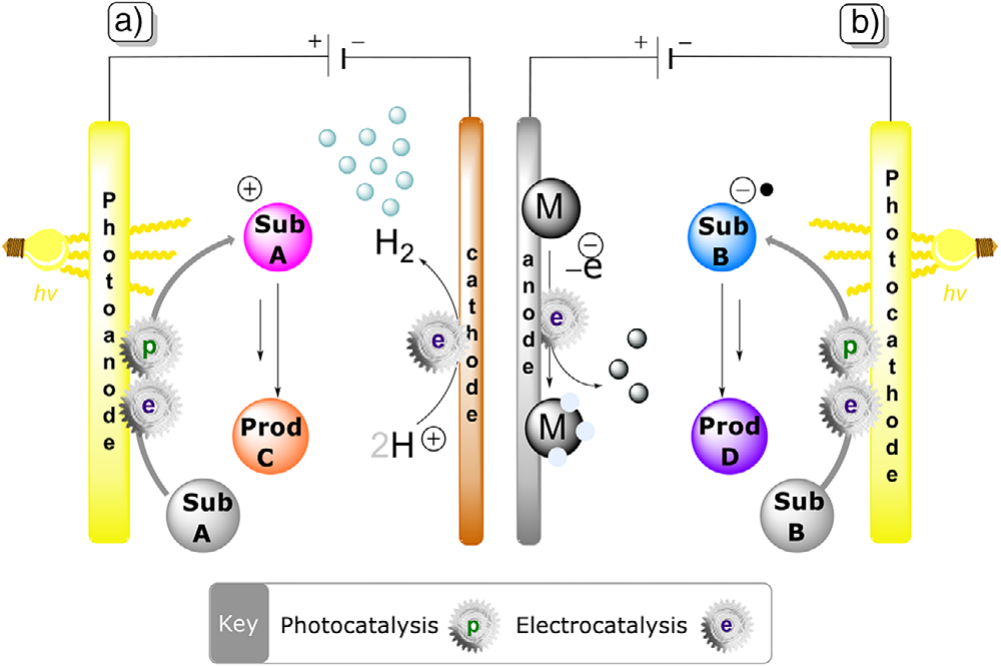
a) General representation of a photoanode oxidizing substrate “A” at its surface and the counter hydrogen evolution reaction. b) General representation of a photocathode, reducing substrate “B” at its surface, and the counter oxidation of a sacrificial metal anode (“M”).

Interfacial photoelectrochemistry (iPEC), or “photoelectrocatalysis,” was defined by the 2011 IUPAC Recommendations as electrochemically assisted photocatalysis, where “the role of the photocatalyst is played by a photoelectrode, often a semiconductor.”^[^ ^58^ ^]^ As such, the term “photoelectrocatalysis” is not appropriate for molecular catalysts activated under PEC conditions in solution.^[^ ^1^ ^]^ In fact, iPEC combines both PRC and EC by replacing one of the electrodes in a typical electrochemical cell with a photoelectrode, which is made of photosensitive materials with band gap corresponding to the visible light photon energy range (400–700 nm, c.a. 3.1–1.8 eV).^[^ ^1^ ^]^ Schematic diagrams of the structures of typical photoelectrochemical cells (PEC cells) for oxidation and reduction are shown in Figure 2.

The working principle behind the PEC cell is analogous to a common electrochemical cell. Upon light irradiation, the photoelectrode material absorbs a spectrum of photons with energy corresponding to or larger than its bandgap. The light absorption material triggers the promotion of an electron in the material from the valence band to the conduction band. This creates an exciton, i.e., a bound electron‐hole particle. The excited electrons and holes are the required species to perform redox reactions on organic molecules. Therefore, to suppress recombination of the carrier pair and acquire as many electrons and holes as possible with the photoexcitation, a constant potential, often also named as an “external bias,” is applied across the cell to facilitate a constant flow of electrons from the photoelectrode to the counter electrode. This increases the rates of desired reactions and can potentially influence downstream reaction pathways.

The counter electrode typically constitutes a metal (such as Zn and Pt) and usually does not directly participate in the target organic transformation. This, however, does not mean that the counter electrode is passive and no reaction will occur on it. iPEC consists of redox processes, which means that reduction‐oxidation reaction pairs must occur simultaneously. While the organic transformation reactions of interest are typically driven by the photoelectrode, in some situations the reaction at the counter electrode can provide useful products, such as H_2_ from hydrogen evolution reaction (HER) or O_2_ from the oxygen reduction reaction (ORR). However, these products are only useful if collected, which is often not the case for iPEC reports that target organic synthesis. This means that half of the PEC cell efficiency is lost to the gas evolution reactions. On the other hand, the counter electrode can be sacrificial, which is frequently seen for anodes in reductive iPEC reactions. This poses additional problems, such as unwanted deposits of counter electrode material on the working electrode, which hinders reusability, reproducibility, or causes clogging of frits/membranes in divided cells.

A reference electrode can be present (should be present) in PEC cells. There are multiple reference electrodes available for aqueous electrolytes, such as the reversible hydrogen electrode (RHE), standard hydrogen electrode (SHE), saturated calomel electrode (SCE), and silver chloride electrode (Ag/AgCl). Voltages recorded between different electrodes can be converted via the Nernst equation, thus offering investigators flexibility in the choice of RE. However, when it comes to organic systems, not all of the reference electrodes mentioned above will function properly. As an example, silver chloride electrodes are problematic to implement into an organic system since such electrodes require a silver salt or potassium chloride to be present; these are insoluble in organic solvents. While an organic soluble silver salt can be added into the organic system, volatility of the organic solvents may cause the potential of the reference electrode to change with time.^[^ ^59^ ^]^ Alternatively, a pseudo‐reference electrode made of a noble metal wire is usually used,^[^ ^60^ ^]^ compounding with an internal reference compound with known redox potential that is soluble in organic solvents, such as ferrocene. This allows the potential to be monitored without the necessity to introduce insoluble ionic metal salts into the organic electrolyte.

### 2.1 Fabrication of Photoelectrodes

There are numerous ways to fabricate semiconductor photoelectrodes, such as chemical vapor deposition (CVD), doctor blading, and thermal procedures. The aim of this section is to provide synthetic chemists with a high‐level overview of the different techniques used to prepare photoelectrodes, comparing and contrasting methods’ advantages and disadvantages. **Note**: In this section, “substrate” shall refer to the sample/surface upon which the semiconductor is attached. To avoid potential misunderstandings, the target molecule in an organic synthetic reaction (see other sections) will not be referred to as “substrate” but as “starting material” or “reactant.”

#### 2.1.1 Chemical Vapor Deposition

**Figure 3:**
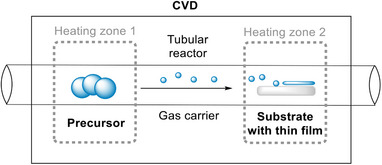
Chemical vapor deposition reactor with two heating zones.

Chemical vapor deposition (CVD) is a vapor‐based technology to deposit thin film materials onto various substrates. In a typical thermal CVD experiment, the thin film precursors and the target substrate are placed into a low‐pressure tubular reactor with two or more separate heating zones that are controlled independently^[^ ^61^ ^]^ (Figure 3). Precursors are sublimated or decomposed under tight temperature, pressure, and gas flow control and transported to the surface of the substrate via a gas‐phase carrier.^[^ ^62^ ^]^ The precursor molecules are physically or chemically adsorbed onto the substrate surface. At a temperature usually higher than that used for sublimating the precursor, chemical reactions take place on the surface of the substrate to form an extensive bonding network, thus constructing a functional thin film. Of note in CVD is that the properties of the thin film material are not necessarily the same as that of the original precursor, which differentiates this technique from physical vapor deposition (PVD) techniques. Compared to PVD techniques, CVD techniques are applicable to produce a wider range of photoelectrode materials, especially organic semiconductors. In PVD, the chemical composition of the precursor is retained after the thin film deposition. However, since most organic semiconductors are polymers themselves, making such material requires several polymerization steps to occur on the substrate in order to obtain the semiconductor thin film. CVD techniques, on the contrary, allow the precursors to react on the substrate surface, and thus the chemical composition and structure of the deposited coating differ from that of its precursors.^[^ ^63^ ^]^

There are further advantages of CVD techniques. It is i) solvent‐free, ii) controllable in purity and reproducibility thanks to the tight control over the deposition parameters, and iii) binder‐free, which eliminates the use of fluorinated organic compounds such as PTFE. CVD also creates thin films with uniform thickness across large substrates regardless of their shape, which allows the topography of substrates to be preserved.^[^ ^64^ ^]^ This is of particular importance for structured surfaces intended for surface‐sensitive applications and even to conformally coat bulk materials. However, the highly conformal deposition of thin films by CVD requires a careful choice of the target substrate, which needs to be stable under the deposition conditions (e.g., temperature). This constraint limits the choice of transparent and conductive substrates available. Furthermore, since the CVD creates a conformal coating on top of the target substrate, the surface area of the resulting thin film is approximately the same as the one of the original substrate, limiting the available surface for the conversion reaction. To overcome this problem and get a photoelectrode with high surface area, a porous electrode material would provide a suitable solution. Lastly and perhaps most importantly, the precursor(s) and the final deposited material have to have a good adhesion onto the surface of the substrate for adsorption and film growth. Crystalline substrate may also affect, in a positive or negative way, the growth of the film, which can affect the film homogeneity and the crystalline structure of the material deposited.^[^ ^65^ ^]^

#### 2.1.2 Solution‐Based Methods

**Figure 4:**
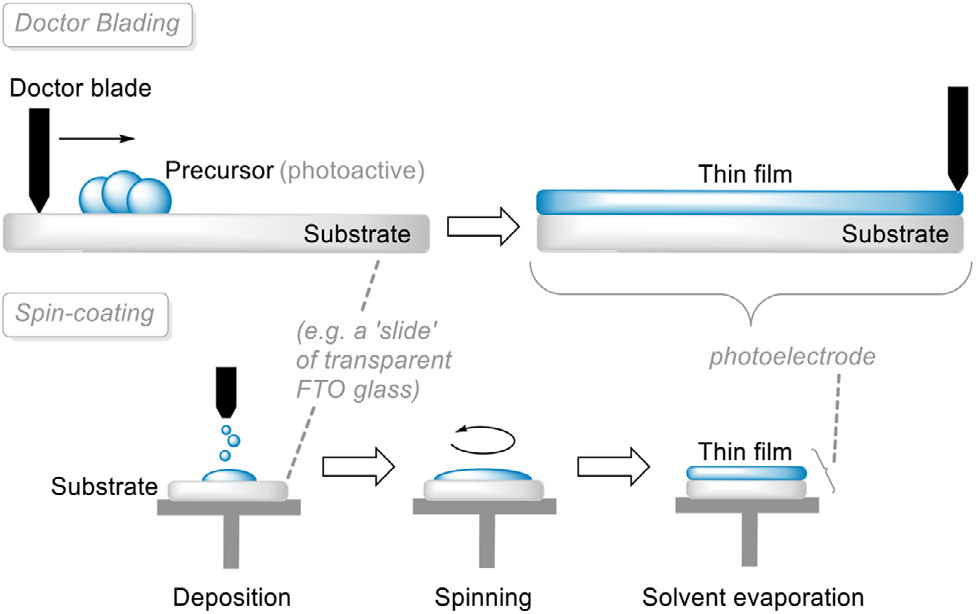
Schematic representations of formations of thin films by doctor blading (top) and spin‐coating (bottom).

Besides vapor‐based thin film deposition techniques, solution‐based methods can be used to cast thin films over surfaces. One of the most common is doctor blading.^[^ ^66^, ^67^ ^]^ In a typical case, a solution containing the thin film precursor is first cast onto the surface of the substrate. A blade then runs through the substrate surface, casting a film usually in the micrometer range thickness (Figure 4, top). It is a simple process that enables to coat even large surfaces using dispersions and solutions.

However, certain disadvantages prevent the mass introduction of doctor blading in semiconductor photoelectrode production. First, a flat surface is required for the process, which means that more complex surface structures and features may not be tolerated. Moreover, the quality of the resulting thin films heavily depends on the preparation of the dispersion liquid. Significant variations in terms of viscosity and homogeneity can arise between different batches of dispersion, which has profound effects on the reproducibility of the casting, especially regarding the uniformity of dispersion spreading and thickness of the resulting thin films. These could potentially alter catalytic activity and thus yields and conversions of the reactions, which is not ideal for scientific investigation.

Spin‐coating^[^ ^68^, ^69^ ^]^ is another example of a commonly used solution‐based thin film deposition technique. Like doctor blading, a solution/dispersion containing the thin film photosensitizer is first cast onto a flat sample, such as a glass slide or silicon wafer. The difference is that the thin film is formed by spinning the sample, causing the solution to be spread evenly on the substrate due to the centrifugal effect. After spreading the solution, the sample stage continues to spin in order to evaporate the solvent from the solution, leading to a solid thin film (Figure 4, bottom).^[^ ^70^ ^]^

The major advantage of spin‐coating is the ease of control over the thin film thickness, which is adjusted by modulating the concentration of the precursor solution and the rotation speed of the sample stage. Owing to the simplicity of the technique, it is routinely used by scientists to create a wide variety of coatings on substrates, such as BiVO_4_/WO_3_ thin films on FTO glasses^[^ ^71^, ^72^ ^]^ or cuprous oxide thin films for photoelectrochemical reactions,^[^ ^73^ ^]^ and polymer thin films.^[^ ^74^, ^75^ ^]^ Nevertheless, spin coating comes with notable disadvantages. First, the material economy of spin‐coating is very low; usually only 2%–5% of the material cast ends up in the formation of thin films.^[^ ^74^ ^]^ Moreover, the geometry and dimensions of the sample are limited: only flat and rigid substrates with relatively small dimensions can be used. Specifically, in the case of polymer thin films, the aforementioned techniques (drop casting, doctor blading, and spin‐coating) may suffice to prepare photoanodes used in aqueous electrolytes due to the organic polymer semiconductors being insoluble in water. However, when used in organic reactions, risks of solubilization of the film on the photoelectrode are notable.

**Figure 5:**
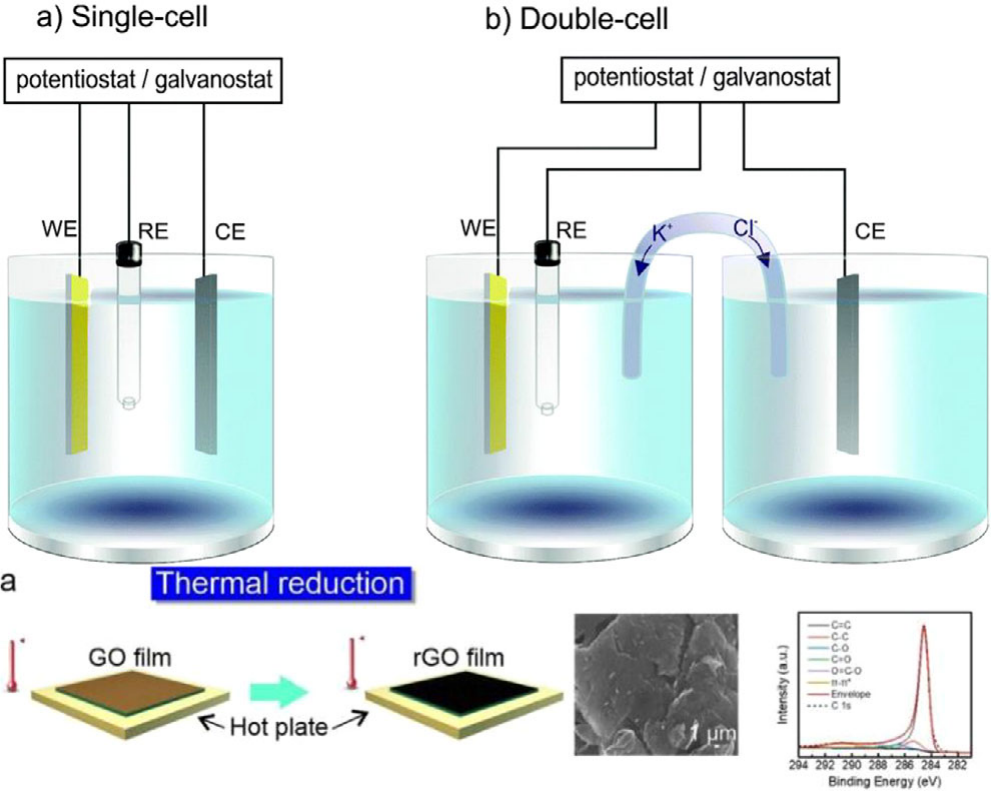
Schematic representation of the process of (top) electrodeposition to produce photoelectrodes and (bottom) thermal annealing. Reproduced from Refs. [79, 80] with permission from the journals.

In addition to the methods discussed, there are other methods that are commonly used for specific types of electrodes. For example, BiVO_4_ electrodes are commonly fabricated by electrodeposition,^[^ ^76^, ^77^ ^]^ and WO_3_ electrodes by thermal annealing (Figure 5).^[^ ^78^ ^]^ These methods all share their unique advantages and disadvantages, where the most suitable method must be determined on a case‐by‐case basis.

### 2.2 Characterization of Photoelectrodes

The prepared photoelectrodes are typically characterized by X‐ray Photoelectron Spectroscopy (XPS), X‐Ray Diffraction (XRD), Fourier Transform Infra‐Red (FT‐IR) spectroscopy, and scanning electron microscopy (SEM), which is often coupled with energy‐dispersive X‐ray spectroscopy (EDS). XPS provides information about surface elemental composition (a few nanometers deep). XRD allows us to confirm or determine the crystal phase of the semiconductor, or its nanocrystalline or amorphous nature. SEM provides information about morphology (surface texture) of the semiconductor, while EDS informs on the elemental composition of the bulk of the semiconductor (a few hundred nanometers). Given that semiconductors are deposited on conductive substrates (such as FTO and ITO, which are crystalline solids), they are often analyzed by the abovementioned techniques.

The photophysical techniques that are typically employed to characterize the photoelectrodes are UV–vis absorption spectroscopy, photoluminescence spectroscopy, and measurements of photocurrent densities. According to the Beer‐Lambert law, a 200‐nm thick coating of a semiconductor, whose typical absorption coefficient is ∼10^5^ cm^−1^ absorbs 99% of incident photons. Therefore, UV–vis spectroscopy is typically employed in diffuse reflectance (DR) mode. DRUV–vis spectroscopy is used primarily to determine the optical gap of the semiconductor and, as such, choose the appropriate excitation wavelength in the iPEC experiments. Steady‐state photoluminescence spectroscopy allows to characterize the efficiency of radiative recombination of photogenerated charge carriers—photogenerated electrons and holes—which is an undesirable process. Among the two semiconductors, radiative recombination of the photogenerated charge carriers is suppressed more efficiently in the one with lower photoluminescence intensity (or, more accurately, lower quantum yield of fluorescence) under otherwise identical conditions. On the other hand, time‐resolved single photon counting (TCSPC) allows measurement of lifetime of the photogenerated charge carriers. Similar to photoredox catalysis, a longer lifetime is beneficial as it maximizes the probability of the redox reaction, unless the reactant is adsorbed on the photoelectrode surface. This eliminates diffusion as the factor limiting the efficiency of the whole process. For example, amines are adsorbed strongly on TiO_2_. While TiO_2_ does not absorb visible light, a complex of tetrahydroquinoline with TiO_2_ features an absorption band at *λ* ≤ ∼500 nm.^[^ ^81^ ^]^ As a result, photochemical dehydrogenation of tetrahydroquinoline at TiO_2_ proceeds upon irradiation with visible light and is likely to involve excitation of the amine–TiO_2_ complex and not the TiO_2_ alone. Photocurrent densities characterize the response of the semiconducting coating to light. Typically, photocurrent densities are measured by performing the linear sweep voltammetry (LSV) experiments upon consecutive switching on and off of the light source (i.e., under “chopped” light irradiation). This allows assessing the dark current and photocurrent in a single experiment at different bias voltages. The obtained photocurrent densities depend on conditions, such as electrolyte, presence of sacrificial electron donor or acceptor, and concentration of electrolyte and solvent, among others.^[^ ^82^ ^]^ For example, the photocurrent densities of carbon nitride‐based photoelectrodes reach ∼1.5 mA cm^−2^ (0.1 M Na_2_SO_4_, 1.23 V),^[^ ^83^, ^84^ ^]^ which, however, are still lower than the current densities in PEC experiments. This is primarily due to the lower conductivity of typical semiconductors compared to metals and carbon (graphite).

## 3 Interfacial Photoelectrocatalysis: Applications in Organic Synthesis

In this section, selected catalytic applications of iPEC using heterogeneous photoelectrodes are reviewed. **Note**: WE = working electrode, CE = counter electrode, and RE = reference electrode. In all case studies below the photoelectrode is the WE.

### 3.1 Oxidation of Simple Alcohols and Carbonyl Compounds

**Figure 6:**
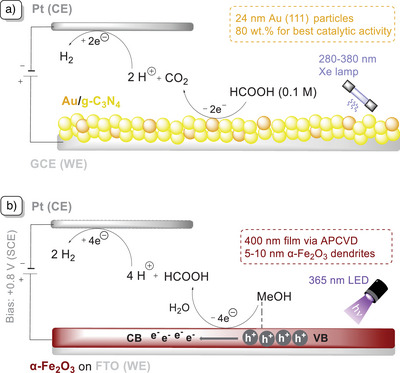
a) PEC oxidation of formic acid by cyclic voltammetry (CV) in an undivided three‐electrode system with Au/g‐C_3_N_4_ nanocomposites on GCE (WE, working electrode), Pt wire (CE, counter electrode), and Ag/AgCl (RE, reference electrode) under Xe lamp (300 W, 250–380 nm) irradiation, HClO_4_ (0.1 M).^[^ ^85^ ^]^ b) PEC oxidation of methanol to formaldehyde in an undivided three‐electrode system with α‐Fe_2_O_3_ (WE), Pt mesh (CE), Ag/AgCl saturated with KCl (RE), NaOH (0.1 M), under 365 nm LED (1 sun) irradiation and at +0.8 V versus Ag/AgCl.^[^ ^88^ ^]^

A pioneering work in iPEC was the oxidation of formic acid, formaldehyde, and ethanol, reported by Xiang and co‐workers (Figure 6a).^[^ ^85^ ^]^ Reactions were conducted with an Au/g‐CN nanocomposite glassy carbon electrode prepared by drop‐casting onto a glassy carbon electrode (GCE) as the photoanode. The presence of Au nanoparticles led to high catalytic activity toward oxidation reactions.^[^ ^86^ ^]^ The authors successfully achieved formic acid oxidation. Current density reached its relative peak at +0.8 V versus Ag/AgCl, indicating that the formation of formic acid is the most rapid at this applied bias. A similar behavior was observed with formaldehyde and ethanol. Different Au loadings were also tested, and differences in current density, resistance, and catalytic performance were evaluated in the order 80 > 70 > 90 > 60 > 50> 20 wt.%. With an increase in Au loading, the density of active sites for formic acid oxidation also increased. However, 80 wt.% of Au loading was chosen for further experiments because an excess of Au nanoparticles led to aggregation.^[^ ^87^ ^]^ The authors claimed that the activity of this composite material was higher than that of pure Au or g‐CN electrodes based on cyclic voltammetry results but did not provide quantitative data on the formation rates and yields of these organic compounds.

Following Xiang's work, the methanol oxidation reaction (MOR) catalyzed by two different photoelectrodes made of Si‐doped α‐hematite (Fe_2_O_3_) and anatase (TiO_2_) under external biases of +0 and +0.55 V versus Ag/AgCl was reported by Durrant and co‐workers (Figure 6b).^[^ ^88^ ^]^ The reaction rate for MOR depends neither on the external bias potential nor the generated photocurrent, but on the surface hole density of the semiconductor. In fact, despite the difference in photocurrents generated, it was shown that the initial rate of MOR on anatase was higher than that of hematite despite having only 30% faradaic efficiency (FE). A similar reaction conducted with hematite had a slower reaction rate, with an FE of 96%. The author attributed the reason to the deeper valence band edge of titania that provides a greater driving force for the transformation of methanol to formaldehyde: for each adsorbed methanol molecule on the metallic center, a proton is released and methoxide is formed. A surface hole then interacts with the methoxide to form a methoxy radical, which then undergoes oxidation to form formaldehyde, injecting an electron into the conduction band of the photoanode in the process.

**Figure 7:**
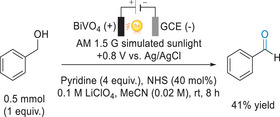
PEC oxidation of benzyl alcohol with BiVO_4_.^[^ ^90^ ^]^

The oxidation of benzyl alcohol (and its derivatives) is one of the most studied reactions in the field of semiconductor organic photoelectrocatalysis. This is due to the fact that the benzylic position of benzyl alcohol is more actively oxidized due to more stable intermediates and aldehyde products. It is thus well‐studied in the field of photocatalysis across various materials and can serve as an indicator of the catalytic performance of a new material. In fact, this reaction is sometimes treated as the “proof‐of‐concept” reaction to benchmark and standardize the catalytic capability of any new photoelectrode material. In 2017, a seminal paper from Berlinguette and co‐workers explored the possibilities of utilizing BiVO_4_ photoelectrodes for alcohol oxidations.^[^ ^89^ ^]^ The BiVO_4_ photoanodes were obtained by spin‐coating a solution of bismuth nitrate hexahydrate and vanadyl acetylacetonate precursors in acetic acid and acetylacetone, followed by annealing and UV curing. With this material and under an external bias of +0.8 V versus Ag/AgCl in 0.1 M LiClO_4_ in MeCN as electrolyte, oxidation of benzyl alcohol to benzaldehyde occurred in 41% yield after 8 h (Figure 7). Under similar reaction conditions, C─H functionalization of cyclohexene and tetralin was also performed and will be discussed later in this review. Pyridine and *N*‐hydroxysuccinimide (NHS), despite being employed in high quantities, had critical rols in the reaction: pyridine acts as proton shuttle to the CE for HER and NHS acts as a redox mediator for the oxidation half‐reaction on the WE (detailed mechanism in Figure 14). The authors noted that, while further optimization is to be made to increase the competitiveness of the reaction and its atom economy, the successful transformation underscored the potential of the iPEC technique in non‐aqueous media.

**Figure 8:**
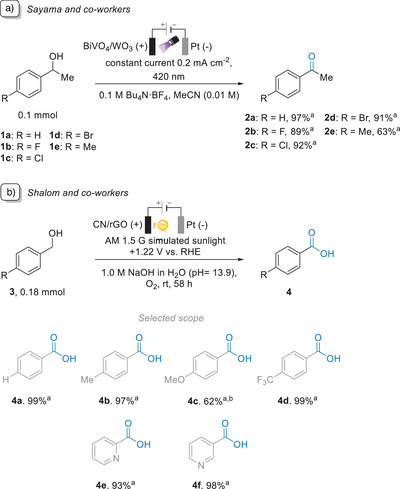
a) PEC oxidation of benzyl alcohol derivatives with BiVO_4_/WO_3_.^[^ ^91^ ^]^ b) PEC oxidation with CN/rGO.^[^ ^92^ ^] a)^Yields determined by GC‐MS or ^1^H‐NMR with an internal standard. ^b)^In case of **3c**, a 38% yield of the benzaldehyde was observed.

Sayama and co‐workers used BiVO_4_/WO_3_ materials as photoanodes for the oxidation of benzyl alcohol derivatives to acetophenones with >99% FE and 63%–97% yield (Figure 8a).^[^ ^91^ ^]^ The PEC oxidations were successfully performed in an undivided cell with a three‐electrode system under constant current (0.2 mA cm^−2^) and 420 nm light irradiation. Furthermore, the oxidation of benzyl alcohol **1a** was achieved in similar yields (>95%) three consecutive times, avoiding any photoanode regeneration process and yielding turnover numbers (TON, i.e. moles of products/BiVO_4_) of approximately 1200. When the scope was extended to *para*‐halogenated (**1b–1d**) and methylated benzyl alcohols (**1e)**, the halogenated species reacted in a similar fashion to **1a**, but **1e** resulted in a lower yield (63%). We speculate that the reason might be due to over oxidations on the *para*‐methyl group. However, in another sense, this could be due to competing deprotonation of the incipient radical cation at that *para*‐methyl position, leading to other undesired products.

Using a different material, Shalom and co‐workers also investigated the oxidation of various substituted benzyl alcohols to benzaldehydes and benzoic acids (Figure 8b).^[^ ^92^ ^]^ The semiconductor in use was carbon nitride (CN) modified with reduced graphene oxide (rGO) to improve conductivity and electron diffusion. Reactions were successfully conducted using the above‐mentioned CN‐based photoanode and a bias potential of +1.22 V versus RHE under AM 1.5 G illumination of 100 mW cm^−2^ and a saturated O_2_ atmosphere. Different benzyl alcohol derivatives were fully converted to benzaldehydes, which further oxidized to benzoic acids with concomitant formation of superoxide (^●^O_2_^−^). The authors showed that reaction time, presence of O_2_ and pH value impact the conversion and selectivity of the reaction. It was discovered that a selectivity of 98%–100% toward benzaldehyde derivatives was observed when the reaction ran for 12–24 h. These derivatives were then oxidized to benzoic acids after 48–58 h with FE > 99%. In the absence of O_2_, conversions of benzyl alcohols were low (39%), as well as the formation of benzoic acids because the limiting concentration of O_2_ in air or (traces in the case of) Ar decreased the formation of ^●^O_2_^−^, a key intermediate in the oxidation of benzaldehydes to benzoic acids. Lower pH values slowed down the reaction, decreasing the conversion of benzyl alcohol, but increased the selectivity toward benzaldehydes (>99%). On the other hand, high pH values (pH > 13) increased the photocurrent and formation rates of benzoic acids. Pure electrocatalytic and photocatalytic experiments were also conducted by switching off the illumination and external bias, respectively, and, in both cases, the yield and conversion were negligible. This confirmed the photoelectrochemical nature of the reaction, which needed both synergistic stimuli for the transformation to occur at a reasonable rate. Notably and contrary to expectations, when substrate **3c** was oxidized, this did not fully convert via its benzaldehyde into its benzoic acid, unlike other benzyl alcohol derivatives where full conversions were observed. The authors contributed this observation to the strong electron‐donating character of the *p*‐OCH_3_ group. While this would normally be expected to promote rates of oxidative transformations, benzylic deprotonation of the benzyl alcohol's radical cation is rate‐determining. The *p*‐OCH_3_ group stabilizes the radical cation of **3c** to benzylic deprotonation, hindering conversion to the benzaldehyde and corresponding benzoic acid.^[^ ^93^ ^]^ The stability of the CN electrodes was characterized by XRD and FTIR after 48 h of iPEC. It was found that in both analytical techniques, the intensities of characteristic CN peaks were decreased with increased reaction time, meaning that structural and morphological changes occurred to the catalyst system during oxidation reactions, resulting in the delamination of CN thin films over time.

With the benzyl alcohol oxidation reaction being performed successfully by various research groups under different conditions, further investigations have focused on optimizing reaction conditions. Bartlett and co‐workers showcased the oxidation of benzyl alcohol with a BiVO_4_ photoelectrode at +0 V versus Fc/Fc^+^ under royal blue LED (448 nm) illumination of 100 mW cm^−2^.^[^ ^94^ ^]^ Specially, the authors included NO_3_^−^ anions in the reaction system, which act as weak bases in MeCN (pK_a_ ∼9). These decrease the photocorrosion of the BiVO_4_ photoanode and serve as radical mediators for the oxidation of benzyl alcohol. On the WE, NO_3_^−^ oxidizes to NO_3_^●^, which reacts with benzyl alcohol through the hydrogen atom transfer (HAT) to give HNO_3_ and benzaldehyde with >90% FE. The required applied potential could thus be decreased by ∼500 mV. Interestingly, in this case, the NO_3_^−^ species were not regenerated at the end of reaction, suggesting they are sacrificial agents.

**Figure 9:**
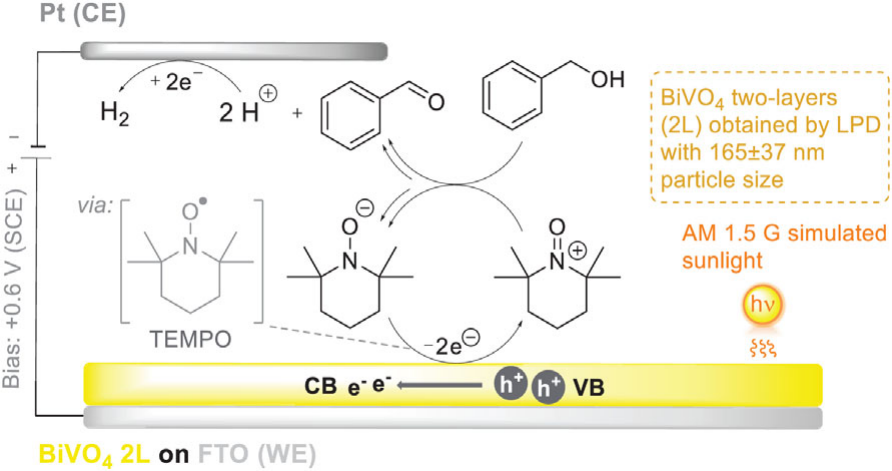
PEC oxidation of benzyl alcohol (25 mM) in an undivided three‐electrode system with BiVO_4_ 2L (WE), Pt wire (CE), TEMPO (5 mM), pyridine (0.1 M), 0.1 M TBAPF_6_, MeCN. Under AM 1.5 G illumination at 1 sun (100 mW cm^−2^) and at +0.6 V versus SCE for 2.5 h.^[^ ^95^ ^]^

Despite being widely studied, photoelectrodes have certain limitations on their efficiency and stability. Taking the semiconductor that is most frequently applied as a photoanode—BiVO_4_—as an example, it was discovered that unmodified BiVO_4_ surfaces are prone to corrosion in aqueous media when a bias is applied. In an attempt to use unmodified two‐layer BiVO_4_ photoelectrodes directly in benzyl alcohol oxidations, Sherman and co‐workers produced a detailed report regarding the role of TEMPO as a hole transfer catalyst in photoelectrocatalysis (Figure 9).^[^ ^95^ ^]^ The two‐layer BiVO_4_ photoanode, obtained by liquid phase deposition (LPD), outperformed the more porous one‐layer surface and provided better insulation from the FTO surface, which generally helps avoid unproductive charge recombination to photochemically formed TEMPO^+^. It was discovered that adding TEMPO dramatically increases photocurrent densities (20%–40%), which contribute to decreasing the required bias potential. The authors evidenced that the formation of benzaldehyde occurs at potentials as low as +0.04 V versus Pt with light irradiation, in contrast to +1.1 V under dark conditions. To obtain a reaction rate similar to a pure electrocatalytic system, an external bias of only +0.91 V versus Pt was required, which accounts for a ∼50% decrease in potential from that of the pure electrocatalytic system (+1.67 V vs. Pt). TEMPO was revealed to be an important component of iPEC alcohol oxidation reactions, serving as both a radical and electron transfer mediator. Its activated (oxoammonium) form, TEMPO^+^, can serve as a hydrogen abstractor, where a hydride transfer occurs from the benzyl alcohol to TEMPO^+^.^[^ ^90^ ^]^

Mas‐Marzá, Fabregat‐Santiago and co‐workers investigated the mechanism behind the photoelectrochemical oxidation of benzyl alcohol by Zr‐decorated BiVO_4_ electrodes.^[^ ^96^ ^]^ Their findings suggested that of the 40% yield of benzaldehyde, over 20% of the products were produced via a mechanism that is independent from the photoelectrode. Another 13.4% of the products were directly related to the photoactivity of BiVO_4_ in the visible light region, without the assistance of external potential. Only 4% of the products were believed to be produced by the PEC activity of the photoanode. From here, it is clear that high photocatalytic activity does not directly translate into excellent activity for photoelectrocatalysis, and researchers have to be aware of unintended contributions toward the reaction yield, which can lead to an overestimation of the photoelectrode performance.

### 3.2 Valorization of Natural Resource Derived Molecules

The bulk/commodity and specialty chemical industries rely heavily on natural resources to provide raw materials for various consumables. Being limited as such, there is often the need to refine or transform compounds harvested into precursors. One common example is the refinement of crude oil into hydrocarbons of different chain lengths for different uses. These refining processes are, however, usually energy intensive and inevitably generate waste. To achieve higher atom economy and energy efficiency, photoelectrocatalysis could be a promising alternative to the traditional refining processes, such as electrocatalysis, by offsetting high potentials needed to engage redox‐inert feedstocks with photon energy. With the ultimate goal of using sunlight as the light source, the energy cost can be further offset, rendering photoelectrocatalysis as a powerful tool to convert waste into usable feedstock for producing other chemicals and consumer products.

**Figure 10:**
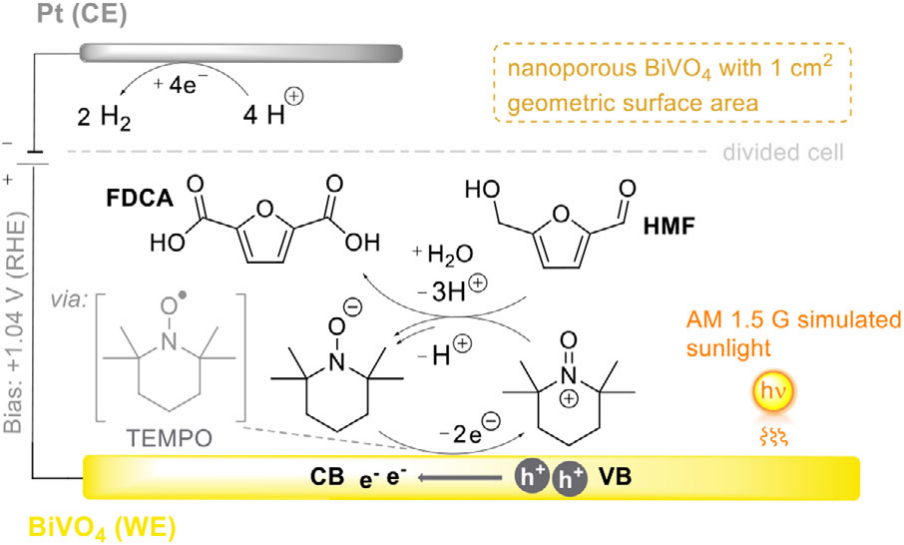
PEC oxidation of HMF (5 mM) to FDCA in a divided three‐electrode system with BiVO_4_ (WE), Pt (CE), Ag/AgCl (KCl 4 M)(RE), TEMPO (7.5 mM), and borate buffer (0.5 M, pH = 9.2). Under AM 1.5 G illumination (100 mW cm^−2^) and at +1.04 V versus RHE for 1 h.^[^ ^97^ ^]^

One notable example of photoelectrochemical preparative decomposition of natural resources was provided by Choi and co‐workers, where they performed the oxidation of 5‐hydroxymethylfurfural (HMF) to various industrially applicable precursors,^[^ ^97^ ^]^ such as 2,5‐furandicarboxylic acid (FDCA), which can be used to produce polymers (Figure 10). Using an *n*‐type BiVO_4_ photoelectrode, TEMPO as a mediator and an external potential of +1.04 V versus RHE, the oxidation of HMF to FDCA was successfully executed with 93% FE. The presence of TEMPO is crucial in this reaction, as it circumvents the need for having a direct electrode‐HMF interaction. When comparing to a pure Au electrode (onset oxidation potential of TEMPO at +1.01 V vs. RHE), the onset potential when using the BiVO_4_ electrode was markedly lower at +0.32 V versus RHE, which is ca. 700 mV lower than the potential needed for HMF oxidation. This is due to the VB edge of the BiVO_4_ photoelectrode being located at approx. +2.4 V versus RHE, which means that the photogenerated holes already possess enough overpotential to trigger the oxidation of TEMPO and thus the cascade of reactions that oxidizes HMF. The role of the external potential is instead postulated to enhance electron‐hole separation in the material to create even more holes for oxidation reactions. As a contrast, the pure electrochemical pathway using a carbon‐felt anode reached comparable yields and FE for HMF only when a high potential of +1.54 V versus RHE was used. This constitutes a decrease of one third in required applied potential when using the iPEC approach.

**Figure 11:**
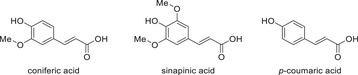
Structure of the possible products of lignin degradation.

King and Chuang recently reported a combined approach to simultaneously perform organic transformations and hydrogen production in one PEC cell.^[^ ^98^ ^]^ The proposed principle is to consume the photogenerated holes on the photoelectrode by breaking C─H and O─H bonds in organic molecules, releasing electrons which would be used to drive the HER. Lignin, a waste biopolymer that is discarded by the paper industry, was chosen as the target material. With a TiO_2_ photoanode, the decomposition of lignin was successfully achieved under different light intensities and current densities. The authors did not specify the identity of the decomposition products of lignin since the target goal was mainly dedicated to using lignin as an electron donor to fuel the HER. However, judging from the characterization results of the reaction mixture given in the literature, it would be reasonable to postulate that the three monomeric alcohols of lignin—coniferyl alcohol, sinapyl alcohol, and *p*‐coumaryl alcohol—were produced by cleaving the lignin molecules. Moreover, it is possible that these alcohols were further oxidized into coniferic acid, sinapinic acid, and *p*‐coumaric acid (Figure 11).

Xiao, Xiong, Liu and co‐workers achieved the oxidation of glycerol into several organic compounds via iPEC, using nanoporous BiVO_4_ photoelectrodes under external biases ranging from +0.8 to +1.2 V versus RHE and at pH values ranging from 2 to 12 under AM 1.5 G simulated solar irradiation.^[^ ^99^ ^]^ It was discovered that the selectivity toward the high‐value products 1,3‐dihydroxyacetone (DHA) and glyceric acid increases with decreasing pH values (up to ∼60% at pH = 2). The production rate of DHA scales with the magnitude of the external potential applied and can reach up to 200 mmol h^−1^ m^−2^ of illumination area.

Following Liu's report, Chiang, Chiu and co‐workers followed up on the investigation,^[^ ^100^ ^]^ focusing on the influence of the crystallographic structure of BiVO_4_ in the photoanode on glycerol selectivity and production. Two different variants of BiVO_4_ photoelectrodes with different dominating crystal surfaces, namely the 010 and 121 facets, were synthesized and investigated for glycerol oxidation under 500 W solar simulator and an applied potential of +1.1 V versus RHE. It was discovered that 010 facet‐dominated BiVO_4_ photoelectrodes enable more efficient adsorption of glycerol and facilitate charge transfer, resulting in higher glycerol oxidation activity and photocurrents than 121‐facet‐dominated BiVO_4_ photoelectrodes. This behavior shows how iPEC depends strongly on mass transfer and is less sensitive than homogeneous PEC to subtle changes in reaction conditions (i.e. behaves more like an electrocatalytic process).

**Figure 12:**
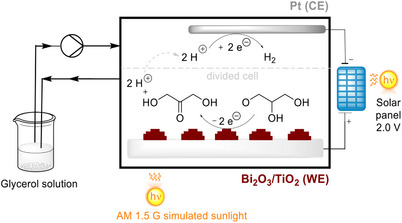
Self‐powering photoelectrochemical system designed for simultaneous production of DHA and H_2_. Oxidation in a divided two‐electrode system with Bi_2_O_3_/TiO_2_ (WE), Pt (CE), Na_2_SO_4_ (0.5 M, pH = 2), and glycerol (0.1 M). Under AM 1.5 G illumination (100 mW cm^−2^) and at a bias of 2.0 V.^[^ ^101^ ^]^

Duan, Li and co‐workers investigated the glycerol oxidation reaction under iPEC conditions using a nanorod titania photoelectrode with bedded Bi_2_O_3_ nanoparticles.^[^ ^101^ ^]^ Employing AM 1.5 G illumination with 100 mW cm^−2^ intensity and under an external bias potential of +1.0 V versus RHE, reaction rates up to 228 mmol h^−1^ m^−2^ of illumination area were obtained, which is slightly higher than Xiao, Xiong, and Liu's conditions.^[^ ^99^ ^]^ Moreover, the selectivity of high‐value organic compounds formed from the oxidation reaction increased to 80% (with >75% DHA selectivity), which is ∼15% higher than what Chiang reported. The elevated reaction rate and selectivity for high‐value organic compounds highlight the benefit of using the hybrid titania‐Bi_2_O_3_ material over nanoporous BiVO_4_. The authors even devised a “self‐powering photoelectrocatalytic system” with a solar panel connected to provide a constant bias potential and the ability to simultaneously produce DHA and H_2_ (Figure 12). In principle, both the external bias and the excitons could be simultaneously provided by sunlight, showcasing the promising potential for practicality and commercialization of iPEC.

### 3.3 C─H Bond Activations and Subsequent Transformations

The major process of harvesting fossil fuels as chemical resources for the industry involves separating them into hydrocarbons of different carbon chain lengths. This is done by fractional distillation, mostly providing fuels but also feedstock hydrocarbons for chemical industry. In the latter case, these feedstock hydrocarbons must be further chemically modified to build complexity before becoming useful. Such transformations oftentimes require hazardous and expensive reagents^[^ ^102^, ^103^, ^104^, ^105^ ^]^ as well as high temperatures and/or pressures.^[^ ^106^, ^107^, ^108^ ^]^ PEC offers alternative methods toward the activation of C─H bonds by diminishing the energetic requirements on reaction conditions, such as the use of molecular PEC to perform direct activations of hydrocarbons.^[^ ^109^, ^110^ ^]^

**Figure 13:**
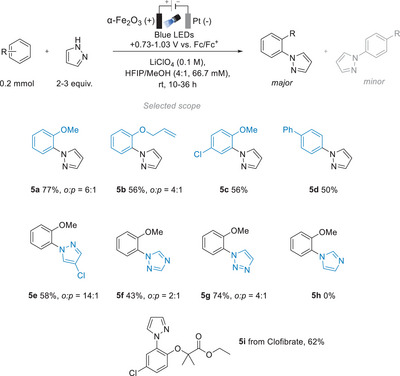
iPEC C─H amination of arenes with α‐Fe_2_O_3_.^[^ ^111^ ^]^

The team of Hu and Grätzel reported iPEC C─H azolations of a wide range of arenes and azoles (up to trisubstituted species), polycyclic aromatic hydrocarbons, and late‐stage pharmaceuticals in various solvents under PEC conditions using α‐hematite (α‐Fe_2_O_3_) as the photoanode and a blue LED light source (Figure 13).^[^ ^111^ ^]^ External potential was set from +0.73 V up to +1.03 V versus ferrocene/ferrocenyl couple (Fc/Fc^+^). With repeated screening of electrolytes, it was discovered that the choice of solvent was the key to enhancing reaction yields. Commonly used organic solvents, such as acetonitrile and 1,2‐dichloroethane, proved to be ineffective, whereas a mixture of 1,1,1,3,3,3‐hexafluoro‐2‐propanol (HFIP) and methanol led to high yields. The amination of a wide range of mono‐, di‐, and trisubstituted benzenes and benzene derivatives was successfully conducted in good to very good (50%–70%) yields. Different nitrogen nucleophiles were also tested. It was discovered that pyrazoles and triazoles were more successful nucleophiles for PEC amination of arenes compared to imidazoles.

Interestingly, compared to direct photoredox and electrochemical oxidations that lead to the *para‐*substituted arenes, under iPEC conditions a high *ortho‐*selectivity was achieved. In order to explain this behavior, the proposed mechanism highlights a possible connection of this selectivity to the solvent HFIP, which may stabilize radical cation intermediates via hydrogen bonding. When hydrogen bonding sites on the arene substituent were not available (e.g., compound **5d**), only the *para* derivative was obtained. The most appealing features of the proposed iPEC method are i) the stability of the photoanode and ii) the high product selectivity (a low abundance of side products). In fact, common side reactions of over‐oxidation of electron‐rich arenes—which are often observed under pure (direct) electrolysis conditions (38% yield of **5a**) —were minimal, increasing the yield to 77% under iPEC conditions. Moreover, the authors noted that the photoelectrode could be reused up to 10 consecutive times without noticeable changes to yields and efficiency.

**Figure 14:**
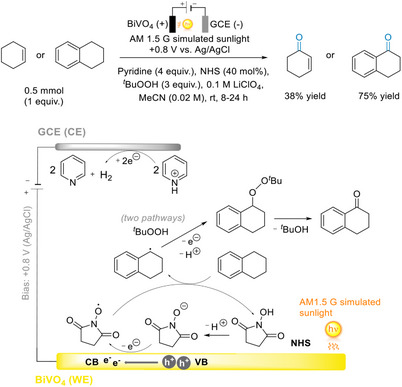
PEC C─H functionalization of cyclohexene and tetralin with BiVO_4_.^[^ ^89^ ^]^

In the same paper as their proof‐of‐concept reaction with benzyl alcohol, Li and Berlinguette reported the iPEC oxidation of cyclohexene and tetralin to cyclohexenone and 1‐tetralone using a BiVO_4_ photoanode, which was previously synthetically challenging (Figure 14).^[^ ^89^ ^]^ In addition to the reactants and conditions mentioned above, an external source of oxygen, *tert*‐butyl hydroperoxide (*t*‐BuOOH), was employed for the formation of *t*‐Bu peroxide‐substrate adduct. Constructive results were given, in which the oxidation of cyclohexene afforded a 38% yield of cyclohexanone after 8 h, and a 93% conversion of tetralin to 1‐tetralone (75% yield) was observed after 24 h. A notable advance in this report was the decrease in required external bias to +0.8 V versus Ag/AgCl. In the absence of light, the electrocatalytic transformation under the same electrolytes and conditions required a higher potential of +1.8 V versus Ag/AgCl. Additional experiments revealed the critical importance of pyridine as a proton shuttle and NHS as a redox mediator for the oxidation half‐reaction (depicted in Figure 14).

Sayama and co‐workers reported the iPEC dimethoxylation of furan using BiVO_4_/WO_3_ composite electrode under an external bias of +0.1 V versus SHE and under AM 1.5 G illumination (100 mW cm^−2^).^[^ ^112^ ^]^ With a pure BiVO_4_ electrode, the dimethoxylated product was obtained with 74% FE but with the composite (BiVO_4_/WO_3_) electrode, the FE rose to >99%. Other semiconductor electrodes, such as TiO_2_ and WO_3_, which are more potent photooxidizing materials, gave only negligible amounts of desired products with FE < 1%, as presumably the oxidation power of the photogenerated holes was too high and oxidation of solvent and methoxylated products predominated. To prevent oxidation of methanol and other side products, a Br^+^/Br^−^ system, proven useful as a mediator in pure electrocatalytic conditions, was employed.^[^ ^113^ ^]^ Here, the role of the Br^+^/Br^−^ ion pair was investigated in detail, where different concentrations of Et_4_N·Br were added into the reaction mixture. The authors reported that no methoxylation product was detected without the presence of Et_4_N·Br, and when Br_2_ was used instead of Et_4_N·Br, the reaction proceeded poorly. The best FE was reached when 10 mM of Et_4_N·Br was added. Interestingly, while higher concentrations of Et_4_N·Br were detrimental to FE, adding a co‐electrolyte (such as Et_4_N·BF_4_) increases the FE to over 99%. The authors speculated that the co‐electrolyte is involved in stabilizing the cationic species generated from furan. The authors also claimed that Br^−^ ions in the reaction mixture were first oxidized by the photogenerated holes to Br^+^‐like species, which then react rapidly with furan and methanol to form the methoxylated products. Subsequently, the same group reported the activation of C─H bonds of cyclohexane (397 kJ mol^−1^),^[^ ^114^ ^]^ which are known to be more inert than other bonds such as C─Cl (339 kJ mol^−1^) or C─Br (289 kJ mol^−1^) and so are more challenging to activate.^[^ ^113^ ^]^ The iPEC oxidation of cyclohexane into cyclohexanol and cyclohexanone (KA oil) was successfully performed under simulated solar light irradiation (100 mW cm^−2^) using a WO_3_ photoanode and an external bias of +2.0 V versus the counter electrode (CE). Upon inspection, it was discovered that the transformation requires the presence of oxygen in the system, as the reaction yielded only a negligible amount of cyclohexanol or cyclohexanone when performed under an atmosphere of N_2_. Furthermore, the proportion of cyclohexanone increased with reaction time, confirming that cyclohexanone is formed from cyclohexanol as an intermediate. The WO_3_ electrode could be reused up to five times without need for reactivation.

**Figure 15:**
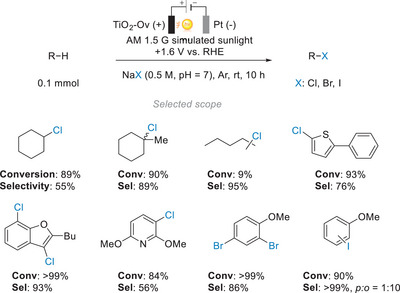
PEC C─H halogenation of hydrocarbons with oxygen vacancy‐rich TiO_2_.^[^ ^115^ ^]^

Duan and co‐workers expanded the scope of PEC C─H functionalization reactions by investigating the possibilities of halogenation through iPEC (Figure 15).^[^ ^115^ ^]^ Several metal oxide semiconductors, including TiO_2_, WO_3_, BiVO_4_, and ZnO, were used to transform cyclohexane into chlorocyclohexane under oxidative conditions. It was discovered that among these selected metal oxides, TiO_2_ had the highest catalytic performance. In order to effectively harvest the solar spectrum, oxygen vacancies were introduced into the TiO_2_ photoelectrodes (“TiO_2_‐Ov”), as these vacancies enhance i) charge separation, ii) absorption in the visible light region, and iii) adsorption of halide ions.^[^ ^116^ ^]^ Halide ions are subsequently oxidized by the photogenerated holes and form radicals or their diatomic species that then react with hydrocarbons. A broad scope of aromatic and aliphatic hydrocarbons was tested for the halogenation reaction using the modified TiO_2_ electrodes in a divided cell system with an external bias of +1.6 V versus RHE and under AM 1.5 G illumination (100 mW cm^−2^) for 10 h. The conversions generally exceeded 80%, with a few being lower than 25% presumably due to steric hindrance or volatility of the reactants. The authors also designed a self‐powered photoelectrochemical system that uses NaCl within seawater as its halogen source and a solar panel to provide a constant bias, producing chlorocyclohexane from cyclohexane at a rate of 412 µmol h^−1^ while simultaneously producing 0.38 mmol of H_2_ per hour of operation.

**Figure 16:**
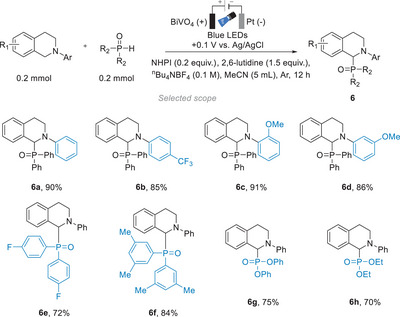
PEC P─H/C─H cross‐coupling reaction with BiVO_4_.^[^ ^117^ ^]^

Wu and co‐workers devised a P─H/C─H cross‐coupling reaction to form a C─P bond,^[^ ^117^ ^]^ as a model reaction to showcase the possibility of using iPEC in the synthesis of organophosphorus compounds that hold important roles in the fields of agrochemicals, materials chemistry, and biochemistry. With a BiVO_4_ photoelectrode under an external bias of +0.5 V versus Ag/AgCl and under blue light irradiation, the proposed reaction (Figure 16) gave a 39% yield of **6a** after 12 h. Upon the addition of a redox mediator, *N*‐hydroxyphthalimide (NHPI), not only could a much lower external bias be used (from +0.5 V vs. Ag/AgCl under pure electrocatalytic conditions to +0.1 V vs. Ag/AgCl under iPEC conditions), but the product yield also increased remarkably from 39% to 90%. The authors proposed the reaction first begins with the reactant being oxidized to a radical cation by the holes generated from BiVO_4_, followed by the deprotonation of NHPI by 2,6‐lutidine. The deprotonated NHPI species then oxidizes also on the surface of the photoanode to give the phthalimide‐*N*‐oxyl (PINO) radical, which reacts with the reactant radical cation to form an iminium ion intermediate. An alternative mechanism could involve electrogenerated PINO acting as a HAT activator of the *N*‐aryl tetrahydroisoquinoline's activated benzylic position, affording a benzylic radical that undergoes facile anodic oxidation to the iminium. Last, the diphenylphosphine oxide reacts with the iminium intermediate to afford the product.

**Figure 17:**
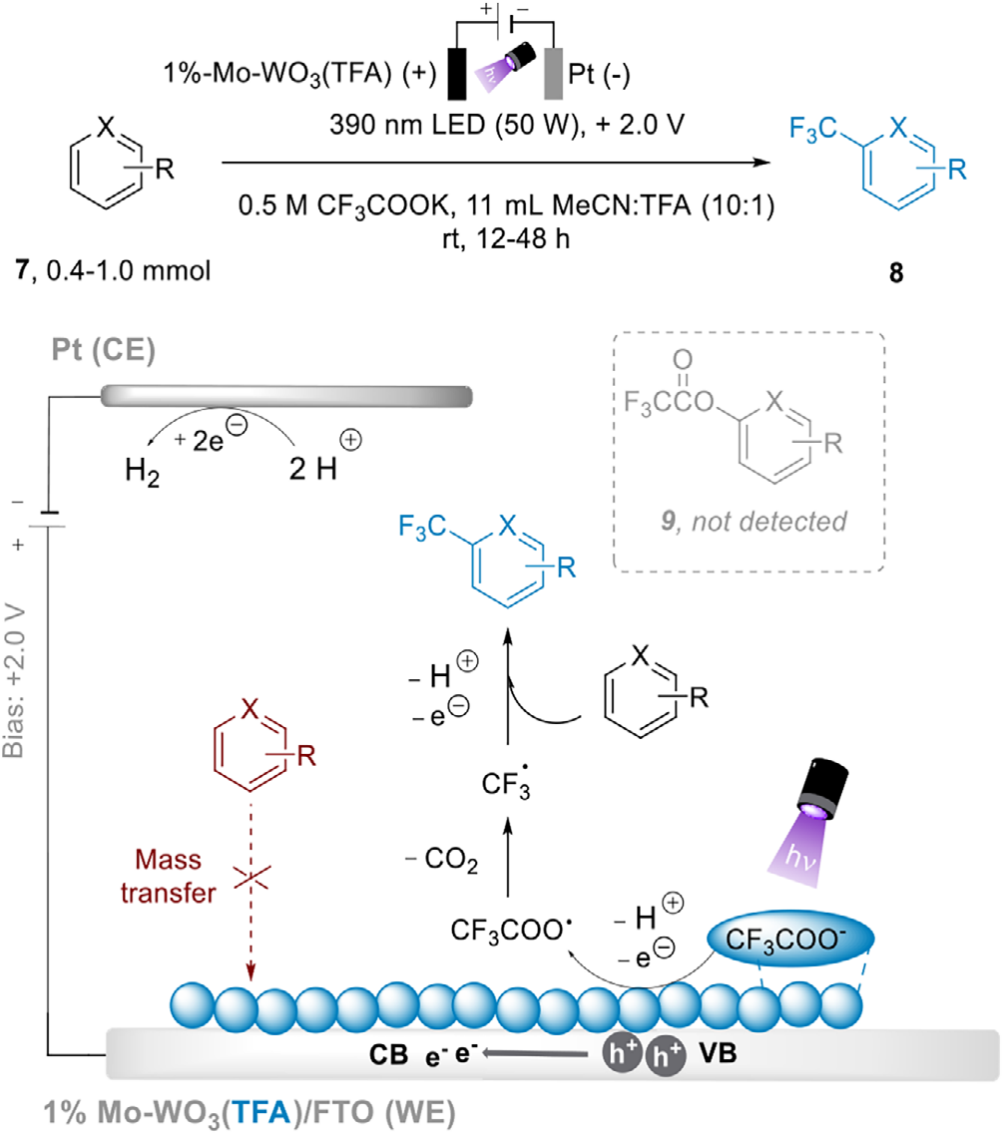
Trifluoromethylation with ion‐shielded Mo–WO_3_.

Chen and co‐workers presented a trifluoromethylation of substituted aromatic compounds under iPEC conditions, using 390 nm irradiation (input power of 50 W) and an external bias of +2.0 V versus SCE (Figure 17).^[^ ^118^ ^]^ The photoelectrode used in this report is composed of molybdenum‐doped WO_3_ thin films on FTO substrate, obtained via hydrothermal synthesis followed by thermal annealing. 1,3,5‐Trimethylbenzene was chosen as the initial reactant to optimize reaction conditions, where a 69% yield was recorded. The scope was then expanded to include various di‐ and trisubstituted benzenes and heterocycles, with yields ranging from 43% to 82%. Notably, with the iPEC approach, side product **9** was not detected, and the selectivity for product **8** was 100%. Other PEC methods employing molecular photoelectrocatalysts such as 9‐mesityl‐10‐methylacridinium ([Mes‐Acr]^+^), 2,3‐dichloro‐5,6‐dicyano‐1,4‐benzoquinone (DDQ), and trisaminocyclopropenium ([TAC]^+^)—and potentials higher than the iPEC method (+3.33 V vs. SCE in the case of [TAC]^+^)—yielded **9** as the only product. It was discovered that the chemoselectivity of the iPEC method was possible due to a layer of CF_3_COO^−^ forming on the surface of the photoanodes, which prevents the direct oxidation of reactant **7** and subsequent coupling of **7** with CF_3_COO^−^, forming side product **9**. The Mo‐doped WO_3_ thin film electrodes showed some stability over time, where a yield of >60% was still obtained at the 4th use. Further investigation revealed that the WO_3_ lattice eventually collapsed after the reaction and led to activity loss. The authors modified their synthesis procedure to replace hydrochloric acid with trifluoroacetic acid, and the resulting electrode showed excellent reusability and gave a yield of >60% for more than 300 h in a continuous reaction.

iPEC allows redox transformations via a photoelectrode in a uniquely selective way, without necessarily requiring an exogenous sacrificial external oxidant. Moreover, this showcases the wide range of possibilities of iPEC as a “greener” method to prepare and functionalize organic molecules.

### 3.4 Comparison between Molecular PEC and iPEC

Although molecular homogeneous PEC and iPEC are two techniques that can be deployed to effectively perform challenging organic transformations, there are notable differences between them. From these differences arise distinctive advantages and disadvantages for each technique, summarized in Table 1 below. The biggest advantage of molecular homogeneous PEC compared to iPEC is its ability to engage a wider redox range of reactants. Taking the amination reaction of arenes as an example, Hu and co‐workers could only achieve amination of activated electron‐rich arenes via iPEC,^[^ ^111^ ^]^ while the groups of Lambert^[^ ^119^ ^]^ and Barham^[^ ^120^ ^]^ were able to engage electron‐neutral and even electron‐deficient arenes using homogeneous PEC by compiling electrochemical redox and photon energies in a molecular catalyst. A comparison can also be made for the work of Mo, Xuan, and co‐workers, where a similar molecular PEC approach by Lum, Wu and co‐workers^[^ ^121^ ^]^ could engage even more electron‐poor (hetero‐)arenes. The main problem lies fundamentally in the concept of photoexcitation. The redox power of semiconductor materials is tightly linked with their band gap; a semiconductor with a wider band gap can engage a wider range of reactants, as it can achieve higher redox potentials. This, however, comes with the penalty of moving to the UV region for light irradiation, since photons of higher energy are required to generate excitons. On the other hand, a semiconductor with a narrower band gap can be photoexcited with photons in the visible light spectrum, yet this leads to lower photocurrents, and the resulting potential is not enough to engage more inert reactants.

**Table 1:** Summary of the advantages and disadvantages for molecular PEC and iPEC.

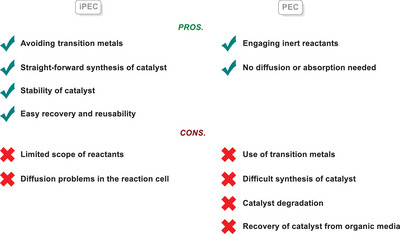

Moreover, a homogeneous catalyst does not rely on diffusion and adsorption of reactants on the catalyst surface, which means that the theoretical reaction rate is not limited by mass transfer.^[^ ^122^ ^]^ However, this also means that the catalysts are difficult to recover due to their high solubilities in organic solvents. Photodegradation of molecular catalysts, relatively complex synthetic access procedures and challenges in catalyst recovery are also problems that thwart the practicality of homogeneous PEC reactions. On the other hand, the heterogeneous semiconductor thin films used in iPEC reactions excel in the ease of catalyst preparation and recoverability. As discussed in Section 2.1, the preparation of semiconductor thin films is relatively simple and straightforward compared to molecular photocatalysts, which makes producing photoelectrodes more economical and feasible for potential scale‐up of PEC reactions. Also, since the photoelectrodes are themselves heterogeneous, recovery of the catalyst can be easily achieved by simply withdrawing the photoelectrode from the PEC cell and washing it appropriately. Another major advantage of using semiconductor thin films for iPEC is the ability to avoid the use of homogeneous transition metal complexes, which are often expensive and have a high environmental impact. The types of electrode material and their differences in surface area also influence the current density (or photocurrent density in terms of semiconductor iPEC).^[^ ^123^ ^]^ Generally, an electrode with higher surface area would lead to lower current density and cell potential, creating a milder reaction environment, benefiting reactions conducted in organic solvents. This is advantageous both in terms of selectivity of reactions and stability of the electrode.^[^ ^124^, ^125^ ^]^

Photoelectrodes used in iPEC methods are usually covered with the semiconductor material only on one side, leading to moderate photocurrent densities; pure electrochemical or other PEC methods usually feature electrodes that are completely submerged in the reaction solution, and all sides of the surface can be fully utilized for redox reactions. A lower current leads to longer reaction times,^[^ ^125^ ^]^ which is not always desirable. This can be mediated by binding surface nanoparticles onto the surface of photoelectrodes or adding co‐catalysts into the reaction mixture. For example, Xiang and co‐workers showed that loading Au nanoparticles on g‐C_3_N_4_ positively influences the current density, up to 80 wt.%.^[^ ^85^ ^]^ However, higher current densities may also increase the temperature of the system and can potentially cause unwanted side reactions, degradation of photoelectrode material, among other issues.

Other major challenges still exist for iPEC, for example, catalytic pathways and active sites of iPEC reactions are harder to predict, proven by the report of Mas‐Marzá, Fabregat‐Santiago and co‐workers on BiVO_4_ that showed even a 10% product yield can be complicated by the combined activation of the photoelectrode by photonic and electrical stimuli.^[^ ^96^ ^]^ Yet, mechanistic aspects and reactivity in molecular PEC reactions can be more straightforward to access because, for example, the electro‐generated radical ion species in those reactions can also be chemically prepared, and its photophysical properties and reactivity studied independently of an electrode surface. Lastly, demonstrations of iPEC for deeply reductive transformations are scarce. This may be due to the fact that photocathodes that absorb solar photons and present high PEC conversion efficiencies are prone to corrosion, which leads to loss of long‐term stability and causes the semiconductor material to be reduced under deeply reductive conditions.^[^ ^126^, ^127^, ^128^ ^]^ These challenges faced by iPEC hinder its application in the field of organic chemistry and synthesis, where homogeneous PRC still gives more benefits over iPEC. Having said this, a strategy involving the merger of molecular photocatalysts and heterogeneous electrodes is gaining rapid attention in recent years toward organic synthesis applications, known as “dye‐sensitized photoelectrocatalysis.”

## 4 Interfacial Dye‐Sensitized PEC: Applications in Organic Synthesis

Interfacial dye‐sensitized photoelectrocatalysis (iDSPEC), a subcategory of iPEC, is gaining more popularity in recent years. The fundamental idea of iDSPEC is to physically immobilize or chemically bind organic dye molecules on the surface of semiconductor photoelectrodes, which serve as the active sites for photocatalytic reactions. The role of the semiconductor material layer here is not to directly engage the organic reactants, but rather to recover the bound organic dyes from their excitation states by electron transfer to complete the redox cycle. Fabrication of the dye‐loaded photoelectrodes is similar to that of the non‐dye‐loaded ones, with the semiconductor layers being prepared by the methods introduced in Section 2.1; the dyes are then loaded onto the semiconductor layers by adsorption,^[^ ^129^ ^]^ forming bonds with the surface groups of the semiconductor,^[^ ^130^ ^]^ or casting.^[^ ^131^ ^]^

The concept of iDSPEC has long been in use for reactions such as water splitting,^[^ ^132^ ^]^ CO_2_ reduction,^[^ ^133^, ^134^ ^]^ and HER^[^ ^135^, ^136^ ^]^ and has proved itself to be effective in those scenarios. Thus, like in iPEC, researchers increasingly sought to perform organic transformations using iDSPEC with various material composites. In the following sections, the applications of iDSPEC in organic transformations are covered to serve as a comparison to existing iPEC techniques. More information on the well‐established methods to use iDSPEC in water splitting or HER can be found in the above‐cited reviews that provide excellent coverage on the respective topics.

### 4.1 Oxidation of Simple Alcohols and Carbonyl Compounds

**Figure 18:**
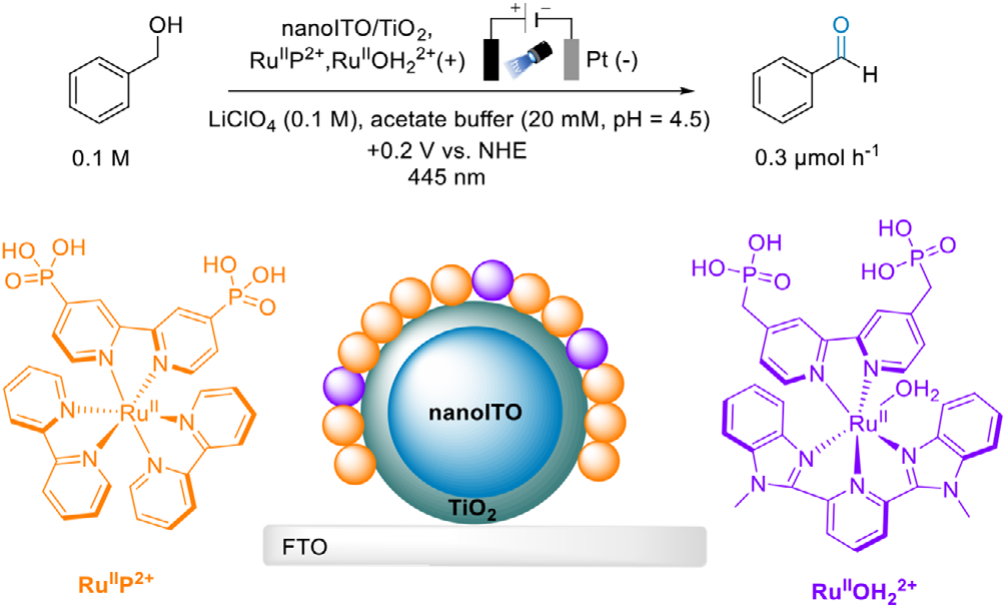
iDSPEC oxidation of benzyl alcohol with nanoITO/TiO_2_.^[^ ^130^ ^]^

An early attempt of using iDSPEC in organic transformations was reported by Meyer and coworkers in 2014^[^ ^130^ ^]^ who developed a multilayer photoanode system (Figure 18). Core/shell nanoparticles with tin‐doped In_2_O_3_ core (nanoITO) and thin layers of TiO_2_ were deposited onto a FTO substrate to form the mesoporous film nanoITO/TiO_2_. Subsequently, this was co‐derivatized with either a ruthenium polypyridyl dye or [Ru^II^(OH)]_2_^2+^, or a mixture of both (Figure 18). Mesoporous TiO_2_ and nanoITO thin films deposited on FTO were also synthesized as a comparison to systematically evaluate the efficiency of the nanoITO/TiO_2_ materials. All three materials were benchmarked using the benzyl alcohol iPEC oxidation under 445 nm light irradiation and under an external bias of +0.2 V versus NHE. Results showed that among the three, mesoporous nanoITO thin films performed the best, with 66% FE and a benzaldehyde production rate of 0.3 µmol h^−1^. The nanoITO/TiO_2_ photoanode could reach a similar rate of production, but FE was only 37%, almost half that of nanoITO.

Reynolds, Schanze and coworkers synthesized another TiO_2_‐based photoanode for iDSPEC oxidation of benzyl alcohol.^[^ ^129^ ^]^ A ligand, 5‐PO_3_H‐2,2′:5′,2″‐terthiophene‐5‐trpy (T3), was first chemically modified with a Ru‐complex and then adsorbed onto the surface of TiO_2_ to form the material TiO_2_–T3–Ru–H_2_O. While the oxidation of phenol and benzyl alcohol by the material was successful under illumination with a 100 mW cm^−2^ visible light source and under an applied bias of +0.2 V, the authors only speculated on the existence of products via analyzing the photocurrent response of the material. No yields, conversions, or FEs of the respective reactions were provided.

**Figure 19:**
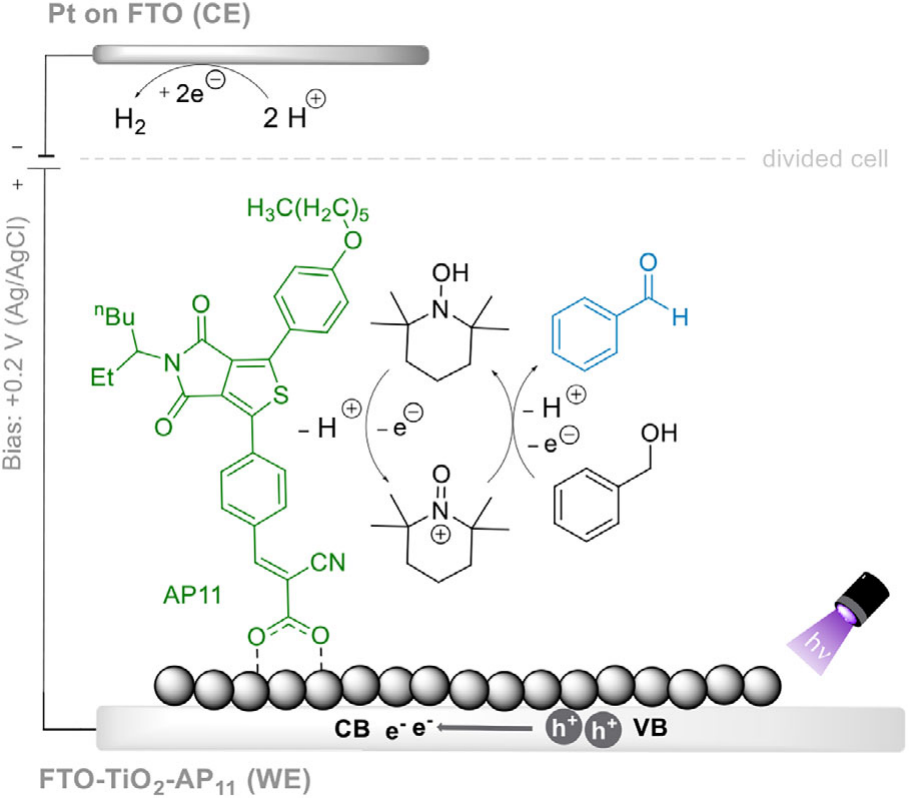
iDSPEC oxidation of benzyl alcohol (0.1 M) in a divided three‐electrode system with (+) FTO‐TiO_2_‐AP11 (WE), MeCN (3 mL), LiTFSI (1.2 M), TEMPO (0.1 M), and C_6_H_5_Cl (0.2 M); (−) FTO‐Pt (CE), MeCN (3 mL), LiTFSI (1.2 M), AcOH (1.0 M), and C_6_H_5_Cl (0.2 M); and Ag/AgCl (RE). Under 100 mW cm^−2^ visible light illumination and at +0.2 V.^[^ ^137^ ^]^

Reek and coworkers have also performed oxidation of benzyl alcohol via mesoporous anatase TiO_2_ thin films loaded with with a metal‐free theinopyrroledione‐based dye (AP11) and TEMPO as an electrochemical mediator (Figure 19).^[^ ^137^ ^]^ Initial photocurrent experiments with a household lamp as irradiation source successfully converted benzyl alcohol into benzaldehyde at an average rate of 1.25 µmol h^−1^ and 74% FE. Control reactions showed that the rate of conversion is negligible without adding the dye or TEMPO to the reaction. When an LED lamp (50 mW cm^−2^, Zahner TLS3) was used as a light source, the rate of benzaldehyde production increased to 4.06 µmol h^−1^ and an FE of 100% was achieved.

**Figure 20:**
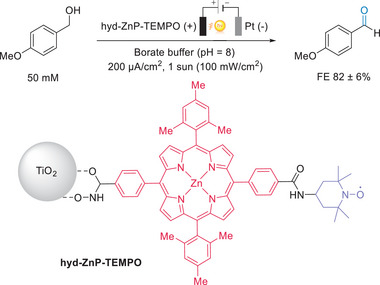
iDSPEC oxidation of anisyl alcohol with hyd–ZnP–TEMPO.^[^ ^138^ ^]^

Odobel and coworkers fabricated a modified TiO_2_ photoanode, used for benzyl alcohol oxidation, where TEMPO was covalently attached to the surface of the photoelectrode via a zinc‐porphyrin (ZnP) sensitizer (Figure 20).^[^ ^138^ ^]^ The breakthrough achieved here was that the catalytic system remained functional in aqueous conditions. With reports showing that alcohol oxidations with TEMPO benefit from basic media,^[^ ^139^, ^140^, ^141^ ^]^ the authors tested the oxidative ability of the synthesized ZnP–TEMPO catalyst on anisyl alcohol (MeO–Ph–CH_2_OH) oxidation to anisaldehyde in a borate buffer (pH 7.5–10) under 1 sun illumination and an average photocurrent density of 200 µA cm^−2^. The same reaction was also performed in acetonitrile with *N*‐methylimidazole to compare the performance of the photoelectrode in an organic environment. Results were surprising, as the catalyst performed the best when reacting in an aqueous environment of pH 8, with FE of 82 ± 6% and high turnover number and frequency. In acetonitrile, the turnover number and frequency achieved were halved, while FE was 76 ± 8%. It was speculated that the high solubility of the ZnP–TEMPO system in acetonitrile leads to its rapid desorption from the TiO_2_ substrate, resulting in lower photocurrent density and hence lower performance. Such behavior could also be seen in aqueous environment (despite the promising FE), where the photocurrent density abruptly drops to 0 in less than 1 h. This confirms that the stability of the zinc porphyrin anchor is a major limitation for the system designed.

### 4.2 Valorization of Natural Resources

**Figure 21:**
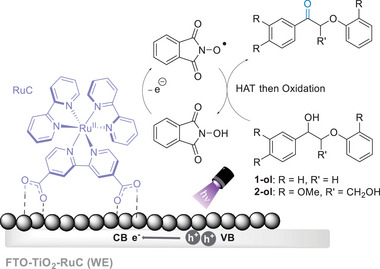
iDSPEC oxidation of lignin‐like structures (2.5 mM) in an undivided system with TiO_2_–RuC (WE), Pt (CE), NHPI/2,6‐lutidine (5 mM), MeCN (10 mL), and TBAPF_6_ (0.1 M). Under AM 1.5 G light source (200 mW cm^−2^) and at +0.75 V versus SCE.^[^ ^142^ ^]^

Leem and coworkers designed an iDSPEC system based on mesoporous TiO_2_ thin films with surface‐bound Ru^II^ complexes coated on top to selectively oxidize benzylic alcohol moieties, with the final aim to depolymerize lignin.^[^ ^142^ ^]^ As shown before in Section 3.2, lignin is a biopolymer where the main constituents are coniferyl, sinapyl, and *p*‐coumaryl alcohol. To extract these alcohols, a selective oxidation of a benzylic alcohol is needed. Here, the authors chose two model compounds to simulate lignin, **1‐ol** and **2‐ol** (Figure 21). Under illumination of an AM 1.5 G light source (200 mW cm^−2^) and an applied bias of +0.75 V versus SCE, the conversions of **1‐ol** and **2‐ol** to their respective ketones were >90% and ∼70%, respectively. To facilitate the catalytic process, 2,6‐lutidine was added to deprotonate NHPI, an HAT co‐catalyst. Upon irradiation, RuC is first excited to RuC*, which then sensitizes TiO_2_ and forms RuC^3+^, which returns to its ground state shortly afterward, leaving behind a hole. Here, authors proposed 2,6‐lutidine deprotonates NHPI, and the resulting species oxidizes to PINO radical, which abstracts a hydrogen atom from **1‐ol** or **2‐ol**. The radical generated by this abstraction goes through another oxidation—either by RuC^3+^ or PINO—to afford the targeted ketones. After successfully oxidizing the two model compounds, a lignin solution was deployed into the same system with identical reaction conditions. A particular moiety on lignin—the α‐OH‐β‐aryl ether—was selected to observe the efficiency of the oxidation. Unfortunately, due to the dark color of lignin solution and the limited accessibility of the hydroxyl group to the catalytic site, only 15% yield (and conversion) was achieved after 24 h. The reusability of the photoelectrode was also investigated, where conversion rate of reactants dropped by about 35% after four reaction cycles (80 h).

In 2022, Reek and colleagues extended the use of their mesoporous anatase TiO_2_ thin films loaded with AP11 from benzyl alcohol oxidation (Figure 19) to glycerol oxidation.^[^ ^131^ ^]^ This time, a special acetonitrile‐based gel layer embedded with TEMPO molecules was cast onto the photoanode to protect it from degradation and to act as a redox mediator layer. Oxidation of glycerol to glyceraldehyde was performed in the anodic compartment of a divided cell, at the gel‐aqueous electrolyte solution's interface. The photoelectrode was illuminated with a white LED (100 mW cm^−2^) and a bias potential of +0.1 V versus Ag/AgCl was applied for 23 h. The conversion reached 100% FE and the rate of production of glyceraldehyde was 2.97 µmol h^−1^. The authors reported that the production rate of glyceraldehyde decreased 10‐fold when TEMPO was not trapped in the gel but instead added to the reaction mixture directly, and the stability of the photoelectrode dropped when the protective gel was not cast onto its surface. As a part of their mechanistic studies, the authors discovered that AP11 dye molecules oxidize TEMPO to TEMPO^+^ at the gel–electrode interface, where the TEMPO^+^ species diffuse to the gelelectrolyte interface and reacts– with glycerol. The authors thus theorized that the reaction needs to “warm up” for a few hours to reach 100% FE, by initial build‐up of TEMPO^+^ in the acetonitrile gel to a steady‐state concentration, which is essential for the oxidation of glycerol to occur at the gel–electrolyte interface.

### 4.3 C─H Bond Activations and Subsequent Transformations

**Figure 22:**
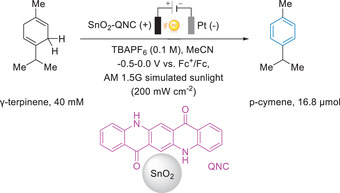
iDSPEC C─H activation of γ‐terpinene in a divided cell with SnO_2_–QNC.^[^ ^143^ ^]^

Sartorel and coworkers reported the possibility to activate C─H bonds of allylic terpene compounds via iDSPEC.^[^ ^143^ ^]^ Quinacridone (QNC), a commercially available and commonly used dye in inks and paints, was chosen as the sensitizer, since it has already been investigated for other non‐organic PEC processes.^[^ ^144^, ^145^ ^]^ With the applied bias of +0.25 V versus Fc/Fc^+^ and irradiation under AM 1.5 G illumination (200 mW cm^−2^, cut‐off filter <400 nm), the QNC‐sensitized mesoporous SnO_2_ electrodes successfully oxidized γ‐terpinene to *p*‐cymene via a HAT mechanism (Figure 22). After 5 h of reaction, 16.8 µmol of *p*‐cymene was produced from 480 µmol of γ‐terpinene, which the authors noted is 32 times higher than the µmol predicted based on the F/mol passed. The authors tentatively attributed this to a photoelectrocatalysis‐initiated radical auto‐oxidation mechanism, where the role of PEC is in initiation. The reaction scope was extended further to other benzylic and allylic compounds. Interestingly, not all the compounds could be transformed. It was discovered that the bond dissociation free energies of the allylic C─H bonds are critical for the reaction, as the limited power of the catalytic system could not abstract H atoms from C─H bonds with dissociation energies higher than 80.5 kcal mol^−1^.

### 4.4 Comparison Between iPEC and iDSPEC

While iPEC and iDSPEC are two techniques with similar principles, there lie certain differences between them, which are summarized in Table 2 below. Advantages and drawbacks of iPEC, such as the ones listed in Table 2, are described in more detail in Section 3.4. On the other hand, advantages and disadvantages of iDSPEC are similar to those of molecular photocatalysts. The preparation of the dye‐loaded photoelectrodes to be used in iDSPEC reactions is, arguably, the most complex amongst PRC and iPEC approaches. Not only must the molecular dyes be synthesized, and the semiconductor thin films deposited onto their substrates, but the dye molecules must also be anchored onto the surface of the semiconductor. This leads to a lower stability of the photoelectrodes. As the organic dyes are usually soluble in organic solvents, they tend to detach from the photoelectrode over time, lowering catalytic performance. However, the use of the organic dyes also presents advantages. Since most of the dyes used are already reported for standalone photocatalytic reactions, the adaptation of these molecular dyes for iDSPEC reactions is relatively more facile. Moreover, as mentioned above in Section 3.4, molecular dye molecules can engage a wider range of reactants for organic transformation, which could potentially also hold true for the dye‐loaded photoelectrodes, given that the stability issue is addressed.

**Table 2:** Summary of the advantages and disadvantages for iPEC and iDSPEC.

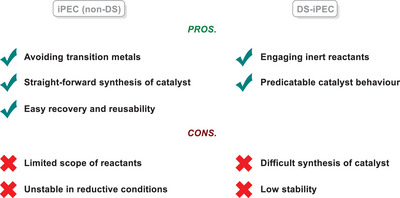

Ultimately, iPEC without dyes may prove more attractive than iDSPEC. As pointed out several times in various reports above, the stability of the dyes immobilized on the semiconductor remains a major issue. Harrison and co‐workers explored the oxidative stability of the immobilized dye molecules on the surface of photoelectrodes, based on a water‐splitting iDSPEC cell.^[^ ^146^ ^]^ It was observed that when an FTO electrode covered in a Ru^II^ complex was soaked in an acidic solution (0.1 M HClO_4_) for 12 h without any irradiation or applied potential, 36% of the ruthenium ligands were lost from the surface. Furthermore, when potential over +1.1 V versus Ag/AgCl was applied, the percentage of Ru(II) complex lost drastically increased to ∼85%. The reason for the steep increase was deduced to be the decomposition and undesired oxidation of the Ru^II^ complex. While Reek and co‐workers attempted to protect the dye molecules by introducing a layer of protective gel, this hindered the diffusion of reactant molecules to the surface of the catalyst, and as reported above, the drawback of such gel is that a certain “warm‐up” period is needed before the reaction can run with 100% FE. Most of the iDSPEC reactions also have lower FEs and yields than their non‐dye‐loaded counterparts, which further lowers the attractiveness of such a technique. However, this is not to say that the idea of iDSPEC is inferior, but rather it should be further integrated with heterogeneous PRC and/or iPEC.

**Figure 23:**
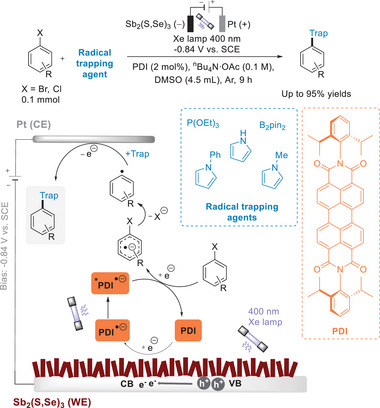
Tandem PEC/PRC for aryl halide functionalization.^[^ ^147^ ^]^

One good example of such an approach can be seen in the work of Wu and co‐workers that combines PRC and PEC to achieve good yields, selectivity, and scope of a reductive functionalization of aromatic halides (Figure 23).^[^ ^147^ ^]^ The authors constructed a system consisting of Sb_2_(S,Se)_3_ layers on FTO as the photocathode and *N,N*‐bis(2,6‐diisopropylphenyl)perylene‐3,4,9,10‐bis(dicarboximide) (PDI) as a molecular photocatalyst. Although this is a partially molecular PEC approach, there are to date no purely photocathodic iPEC examples. Under AM 1.5‐fitted Xe lamp (500 W) irradiation and under an external bias of −0.84 V versus SCE, the coupling of 4‐bromoacetophenone with *N*‐Me pyrrole yielded 89% of the product after 9 h. Switching the light source to 450 nm was also favorable for the reaction and yielded 68% of desired product. The scope of reactants was then expanded and targeted as well as the more challenging to reduce aryl chlorides and late‐stage functionalization. Moreover, it was discovered that the selectivity of the reaction was high and reductively labile functional groups (such as carbonyl, cyano, and sulfonamide) were not reduced. Control reactions with only light gave significantly lower yields compared to iDSPEC conditions, and without PDI, the reaction gave traces of product. Furthermore, differently from König and co‐workers,^[^ ^53^ ^]^ where 8 equivalents of sacrificial NEt_3_ were needed to inject the energy of two photons into PDI, in this coupled PRC/PEC system sacrificial agents are completely avoided.

## 5 Future Perspectives

### 5.1 Carbon Nitride Photoelectrode Materials

As mentioned before, in the field of PEC, most of the attention has been devoted to using metal or transitional metal oxide photoelectrodes such as TiO_2_, α‐Fe_2_O_3_, WO_3_, and BiVO_4_. However, most of these metal semiconductors have inherently low abilities to harvest solar light due to their wide band gaps^[^ ^148^ ^]^ or high recombination losses^[^ ^149^ ^]^ that hinder production of sufficient photocurrents under visible light conditions. Under UV‐ or near‐UV light irradiation, where these metal semiconductors are more effective, there is a palpable risk of direct excitation of the organic molecule reactants in the reaction system, where they also absorb effectively that cause undesirable reactions to occur.

Graphitic carbon nitrides (g‐CNs) are promising alternatives to these metal‐based semiconductors, with advantages coming from environmental and material points of view: the composition elements of g‐CNs are abundant and non‐toxic C and N, meaning that the disposal and post‐processing of degraded g‐CN materials is facile.^[^ ^150^, ^151^ ^]^ While the band gap of g‐CN materials depends on the structure, those with the chemical composition of C_3_N_4_ have a band gap in the range of 2.6–2.9 eV^[^ ^152^, ^153^ ^]^ with absorption in the visible light range, up to 460 nm.^[^ ^154^ ^]^ The material is also thermally stable up to 600 °C and chemically resistant to acids.

#### 5.1.1 Carbon Nitride Photoelectrode Material Synthesis

**Figure 24:**
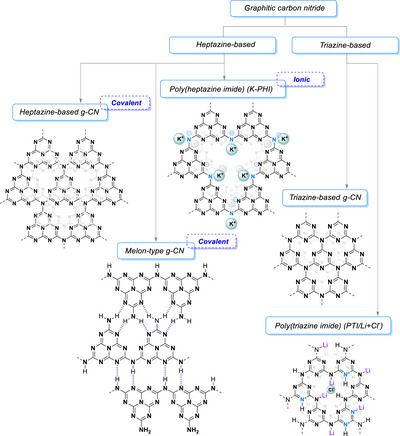
Overview of different structures of g‐CN.^[^ ^163^ ^]^

Several structures of graphitic carbon nitrides with C:N ratio of 4:3 have been reported until now, including melon‐type CN,^[^ ^155^ ^]^ heptazine‐based CN (single crystals were not reported, these are poly(heptazine imides),^[^ ^156^, ^157^, ^158^, ^159^ ^]^ triazine‐based g‐CN,^[^ ^160^ ^]^ and LiCl‐intercalated poly(triazine imide) (PTI/Li^+^Cl^−^),^[^ ^161^, ^162^ ^]^ as seen in Figure 24.

**Figure 25:**
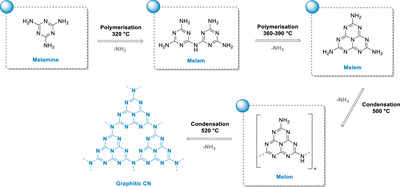
Principal synthetic route of graphitic carbon nitride.^[^ ^150^ ^].^

Among these structures, melon‐type g‐CN and fully condensed g‐CN are the most reported in scientific literature concerning iPEC. Figure 25 shows the principal chemical steps leading toward the formation of these two kinds of g‐CNs. A variety of nitrogen‐rich precursors can be used for the synthesis of this polymer, and often the choice is to use melamine, since it already contains the triazine ring. Formation of g‐CN starts with the dimerization of melamine into melam at ∼320 °C and further into the trimer melem (tri‐*s*‐triazine) at around 360–390 °C. Melem, the melamine trimer, is the repeating unit found in heptazine‐based graphitic carbon nitrides. Final condensation of melem into a full g‐CN polymer occurs at around 520 °C.^[^ ^150^, ^164^ ^]^ Fully condensed g‐CNs, i.e., those featuring tertiary nitrogen atoms as the interlink of the basic repeating units, are present as local structures in g‐CNs.

Owing to their ease of preparation and excellent material properties, the last two decades have witnessed many efforts on utilizing g‐CN as a material for various applications. In fact, g‐CN can be used in photocatalysis and electrocatalysis,^[^ ^154^, ^165^ ^]^ energy devices,^[^ ^148^, ^150^ ^]^ as well as in the fields of optical materials, sensors, actuators, and biomedical materials.^[^ ^166^, ^167^ ^]^ The details of such applications are, however, out of the scope of this review, and are already covered in the reviews cited above.

While g‐CNs feature suitable physical and chemical properties to be applied in the field of photocatalysis, their presence in the field of iPEC is relatively scarce;, mainly featuring in photocatalysis of inorganic transformations.^[^ ^168^, ^169^, ^170^, ^171^, ^172^, ^173^, ^174^, ^175^ ^]^ Two major obstacles hinder the mass assimilation of g‐CN into the field of iPEC: first, a PEC cell features a reactor design that requires the catalyst to be a self‐standing electrode if not deposited onto a conductive substrate, as mentioned in the section above. Since the electron conductivity of g‐CN is inherently low (typically << 1 S cm^−1^)^[^ ^148^, ^176^ ^]^ and anisotropic, a self‐standing g‐CN electrode is not a viable form of photoelectrode. A composite electrode with g‐CN deposited on a conductive substrate is more viable and suitable for PEC applications. The preparation of such electrodes would require the deposition of a homogeneous, conformal, and thin layer of g‐CN with thickness in the range of nanometers on top of the conductive substrates Homogeneity is needed to ensure that the catalytic activity across the electrode area remains constant, and thinness is needed to minimize the disadvantages of g‐CN's short electron diffusion length.

#### 5.1.2 Carbon Nitride Photoelectrode Material Fabrication

There are two main ways to grow g‐CN thin films onto substrates; direct growth and indirect growth. As this review is not dedicated to summarizing and discussing the various methods to produce g‐CN photoelectrodes, the following paragraphs will serve to highlight certain milestones achieved in recent years. A more detailed explanation of the previously reported methods can be found in the excellent reviews published before.^[^ ^148^, ^150^, ^166^ ^]^

Indirect growth methods feature a two‐step process to fabricate g‐CN electrodes: first, the g‐CN is synthesized through conventional methods and then suspended in a solution. The thin film is then deposited via an additional step onto the conductive substrate. This could be spin‐coating or dip‐coating,^[^ ^177^, ^178^ ^]^ doctor blading,^[^ ^71^ ^]^ or electrospinning.^[^ ^179^ ^]^ The advantages of these methods are that both synthesis steps and the deposition steps are well‐established in the scientific community, and thus only require optimizations to fit individual use cases, which makes them more reliable for up‐scaling.^[^ ^166^ ^]^ However, they also have their inherent disadvantages that come with the film deposition method, such as the inability of spin‐coating to coat a large surface due to difficulties in film thinning,^[^ ^76^ ^]^ or the impacts of doctor blading on substrate geometry.

Direct growth methods are methods where the g‐CN thin films are deposited directly onto the substrates. These can be further subdivided into solid/liquid‐phase contact growth and gas‐phase non‐contact growth methods.^[^ ^166^ ^]^ The earliest attempts to coat conductive substrates directly with g‐CN thin films were thermal vapor condensation,^[^ ^180^ ^]^ microcontact printing,^[^ ^168^, ^181^ ^]^ and vapor deposition polymerization. Vapor deposition polymerization was a major breakthrough in the fabrication of g‐CN photoelectrodes to deposit crack‐free g‐CN films on conductive substrates with the ability to control film thickness, first reported by Aida and co‐workers in 2016.^[^ ^182^ ^]^

Giusto and co‐workers developed a method based on CVD that enables homogeneous and conformal deposition of g‐CN thin films, regardless of substrate geometry. Compared to previous methods, this method presents several advantages, including the tight control of thin film thickness and reproducible and steady thin film qualities, by using a two‐zone CVD system in vacuum.^[^ ^61^ ^]^ Precise control of thickness is achieved by adjusting the amount of precursors used for the deposition, with height ranging from a few nanometers to hundreds of nanometers. These resulting thin films can be used in several applications, such as in sodium‐ion batteries^[^ ^183^ ^]^ and as initiators for polymerization^[^ ^184^ ^]^ with tunable thickness. Moreover, the g‐CN thin films produced are of optical quality.^[^ ^185^ ^]^ Not only do these thin films open doors for further applications into fields such as photonics and optical electronic materials, they offer great promise in photocatalysis. Conventionally produced g‐CN thin films do not permit the transmittance of light, which creates constraints in reactor set‐ups. With this particular method, however, the resulting thin films are transparent in nature, which, with appropriate choice of substrates, allows back‐side illumination of the thin films, giving more freedom to reactor designs and setups.

Savateev, Giusto and co‐workers explored the potential of utilizing such g‐CN thin films as photocatalysts in 2021,^[^ ^64^ ^]^ where the thin films were coated uniformly on glass reaction vials. Under 400 nm light irradiation, benzyl alcohol was successfully converted into benzoic acid, achieving ∼90% yield after 24 h. Simultaneously explored was the capability of these CVD‐grown g‐CN thin films in a flow chemistry setting, which achieved near‐full conversion of benzyl alcohol species in less than 2 h, showcasing the ability of these CVD‐grown g‐CN thin film materials to act as photocatalysts. The report from Gschwind and co‐workers further supports this claim, where sp^3^ C─F bond formation experiments were performed with CN‐coated vials and NMR tubes, which enabled them to prove the fluorination mechanism via the development of an in situ NMR setup coupling the NMR‐coated tubes with an optical fiber.^[^ ^186^ ^]^ Eventually, the thin film deposited in this way also enables the development of further in situ spectroscopic characterization. By combining XPS and XAS characterization with in situ illumination, it was possible to depict the critical role of surface interactions and the mechanism of the photocatalytic water splitting reaction using CN thin films.^[^ ^187^ ^]^

Shalom and co‐workers have published extensive works related to the synthesis and use of g‐CN thin films as photoelectrodes in the past decade, where they explored numerous synthetic methodologies throughout. Fabrication methods included indirect growth methods such as dip coating,^[^ ^169^ ^]^ doctor blading,^[^ ^170^ ^]^ as well as direct growth methods such as thermal vapor condensation,^[^ ^82^ ^]^ electrophoretic deposition,^[^ ^172^ ^]^ and even multilayer synthesis by combining different approaches. Most importantly, the roup focused intensely on the modification of carbon nitride materials to suit different photoelectrochemical reactions, such as doping,^[^ ^173^ ^]^ incorporation of other kinds of active catalysts like metal‐organic frameworks (MOFs),^[^ ^174^ ^]^ transition‐metal complexes,^[^ ^175^ ^]^ and rGO.^[^ ^176^ ^]^ While these were mainly applied as photoanodes for inorganic reactions like water splitting or OER, it was also shown that carbon nitride materials can be applied to organic transformations with excellent performances by this group, previously mentioned in Section 3.1.^[^ ^92^ ^]^ Moreover, the photoanodes synthesized by Shalom and co‐workers were also applied in reductive use cases (still as photoanodes),^[^ ^63^ ^]^ which is not commonly seen for carbon nitride materials due to the fact that their band structures favor oxidations.^[^ ^188^, ^189^ ^]^ This helps broaden the potential of carbon nitride materials even further, enabling more different organic transformations.

In conclusion, carbon nitride materials offer promise as a non‐metallic alternative to metal oxide semiconductor photoelectrodes that are currently trending in use, as shown above in previous sections, owing to the intrinsic advantages of being non‐toxic, chemically stable, recyclable, and easily disposed of.

### 5.2 Nitrogen Fixation Reactions

Nitrogen fixation is one of the most important chemical processes that occurs naturally and artificially. Both biological and artificial nitrogen fixation aim to produce ammonia (NH_3_) molecules from atmospheric nitrogen, which is an important precursor to essential molecules such as amino acids. In modern society, ammonia is fundamental for the preparation of fertilizers, pharmaceutical products, and important nitrogen‐containing chemicals such as hydrazine, hydrogen cyanide, and urea. Despite the fact that the global production of ammonia reaches over 150 million tons per year,^[^ ^190^ ^]^ human society relies almost exclusively on the Haber–Bosch process, which is itself highly energy intensive and produces a high footprintmount of greenhouse gases: the process alone contributes to around 1% of the global greenhouse gas emission.^[^ ^175^ ^]^

During the last decade, interest in photo‐ and electrocatalytic methods for nitrogen fixation has been a center of focus in exploring alternative approaches to the Haber–Bosch process. While ammonia has been successfully produced, these methods suffer from i) limited FE,^[^ ^190^ ^]^ ii) such poor turnover/mass production that background ammonia becomes problematic and results in “false positives,” and iii) difficulties in separating products from the reaction mixture.^[^ ^191^ ^]^ Photoelectrocatalysis, combining the advantages of both photocatalysis and electrocatalysis, as well as avoiding the challenging product separation, can be of great use in such direction. Yet, while extensive investigations and reports on the production of ammonia via photoelectrocatalysis are available,^[^ ^190^, ^191^, ^192^, ^193^, ^194^ ^]^ there is a notable deficiency of literature concerning direct fixation routes generating nitrogen‐containing organic molecules directly from atmospheric N_2_. We foresee this as a promising target area for future development and would encourage the community to pursue said direction.

## 6 Conclusions and Outlook

Semiconductor organic PEC is a thriving research field that has benefited from the revitalization of photocatalysis and electrocatalysis, as well as an increasing understanding of semiconductor properties by the scientific community. Compared to photocatalysis and electrocatalysis, photoelectrocatalysis often permits reactions to occur i) at lower applied potentials to the reaction system, ii) in milder reaction conditions, and iii) often at higher reaction rates. However, it is far from suitable to conclude that photoelectrocatalysis could be the ultimate solution for the energy and global warming crisis, as there are still problems to be solved. With the aim of this review being not only to summarize the advancements achieved but also to identify challenges and hence help dismantle hurdles to innovations in the field, it would be beneficial to highlight some of the challenges and prospects.

The first major obstacle is the lack of absorption of the photoelectrodes in the visible light region. As mentioned before, most of the metal‐based materials do not absorb light in the visible light range, except for a few like BiVO_4_ and WO_3_. While they could be replaced by greener and metal‐free materials like graphitic carbon nitride, where its band gap width lies within the visible light range, they are still plagued with challenges in electronic conductivity and high recombination losses. Efforts were already made by scientists to synthesize photoelectrodes with composite materials, as shown above with the hybrid BiVO_4_/WO_3_ and Au‐embedded g‐CN photoelectrodes, which show better absorption in the visible light range and higher catalytic activity. Combined with the idea of an artificial Z‐scheme/Schottky junction, the productivity of PEC can increase significantly, leading to practical industrial applications.

Moreover, the range of reactions that have been investigated via PEC is, compared to photocatalysis and electrocatalysis, relatively small. Most of the reports focus on more standard reactions, such as oxidation of alcohols to carbonyl compounds, due to them being well understood throughout the chemical community. While to say such reactions are of less importance would be a grave mistake, there are still a lot of chemical transformations with industrial significance that are energy intensive and/or rely exclusively on noble metal catalysts that are not in accordance with the principles of sustainable and green chemistry, namely C─C or C–heteroatom coupling reactions, transformation of atmospheric nitrogen into high‐value organic compounds and harvesting of biological feedstocks. These reactions are crucial to the future of humanity, as the time left for our species to reach carbon (and nitrogen!) neutrality is scarce.

Though being a relatively new field, with the scalability and commercialization aspects still outstanding challenges, reports of completely self‐powering photoelectrocatalytic devices are starting to emerge, forecasting a promising greener method to produce various organic molecules required by the industries and the scientific community.

## Conflict of Interests

The authors declare no conflict of interest.

## Data Availability

Data sharing is not applicable to this article as no new data were created or analyzed in this study.
